# Monitoring drug–target interactions through target engagement-mediated amplification on arrays and *in situ*

**DOI:** 10.1093/nar/gkac842

**Published:** 2022-10-03

**Authors:** Rasel A Al-Amin, Lars Johansson, Eldar Abdurakhmanov, Nils Landegren, Liza Löf, Linda Arngården, Andries Blokzijl, Richard Svensson, Maria Hammond, Peter Lönn, Johannes Haybaeck, Masood Kamali-Moghaddam, Annika Jenmalm Jensen, U Helena Danielson, Per Artursson, Thomas Lundbäck, Ulf Landegren

**Affiliations:** Department of Immunology, Genetics and Pathology, Science for Life Laboratory, Uppsala University, Uppsala, Sweden; Department of Medical Biochemistry and Biophysics, Chemical Biology Consortium Sweden (CBCS), Science for Life Laboratory, Karolinska Institutet, Solna, Sweden; Department of Chemistry-BMC, Science for Life Laboratory, Uppsala University, Uppsala, Sweden; Center for Molecular Medicine, Department of Medicine (Solna), Science for Life Laboratory, Karolinska Institutet, Solna, Sweden; Department of Medical Sciences, Uppsala University, Uppsala, Sweden; Department of Immunology, Genetics and Pathology, Science for Life Laboratory, Uppsala University, Uppsala, Sweden; Department of Medical Sciences, Uppsala University, Uppsala, Sweden; Department of Immunology, Genetics and Pathology, Science for Life Laboratory, Uppsala University, Uppsala, Sweden; Department of Pharmacy, Uppsala University Drug Optimization and Pharmaceutical Profiling (UDOPP), Science for Life Laboratory, Uppsala University, Uppsala, Sweden; Department of Immunology, Genetics and Pathology, Science for Life Laboratory, Uppsala University, Uppsala, Sweden; Department of Immunology, Genetics and Pathology, Science for Life Laboratory, Uppsala University, Uppsala, Sweden; Institute of Pathology, Neuropathology and Molecular Pathology, Medical University of Innsbruck, Innsbruck, Austria; Diagnostic and Research Institute of Pathology, Medical University of Graz, Graz, Austria; Department of Immunology, Genetics and Pathology, Science for Life Laboratory, Uppsala University, Uppsala, Sweden; Department of Medical Biochemistry and Biophysics, Chemical Biology Consortium Sweden (CBCS), Science for Life Laboratory, Karolinska Institutet, Solna, Sweden; Department of Chemistry-BMC, Science for Life Laboratory, Uppsala University, Uppsala, Sweden; Department of Pharmacy, Uppsala University Drug Optimization and Pharmaceutical Profiling (UDOPP), Science for Life Laboratory, Uppsala University, Uppsala, Sweden; Department of Medical Biochemistry and Biophysics, Chemical Biology Consortium Sweden (CBCS), Science for Life Laboratory, Karolinska Institutet, Solna, Sweden; Department of Immunology, Genetics and Pathology, Science for Life Laboratory, Uppsala University, Uppsala, Sweden

## Abstract

Drugs are designed to bind their target proteins in physiologically relevant tissues and organs to modulate biological functions and elicit desirable clinical outcomes. Information about target engagement at cellular and subcellular resolution is therefore critical for guiding compound optimization in drug discovery, and for probing resistance mechanisms to targeted therapies in clinical samples. We describe a target engagement-mediated amplification (TEMA) technology, where oligonucleotide-conjugated drugs are used to visualize and measure target engagement *in situ*, amplified via rolling-circle replication of circularized oligonucleotide probes. We illustrate the TEMA technique using dasatinib and gefitinib, two kinase inhibitors with distinct selectivity profiles. *In vitro* binding by the dasatinib probe to arrays of displayed proteins accurately reproduced known selectivity profiles, while their differential binding to fixed adherent cells agreed with expectations from expression profiles of the cells. We also introduce a proximity ligation variant of TEMA to selectively investigate binding to specific target proteins of interest. This form of the assay serves to improve resolution of binding to on- and off-target proteins. In conclusion, TEMA has the potential to aid in drug development and clinical routine by conferring valuable insights in drug–target interactions at spatial resolution in protein arrays, cells and in tissues.

## INTRODUCTION

Analysis of target binding by small drug molecules is critical in drug discovery as it ties interactions with intended targets and unwanted off-targets to clinically relevant pharmacology (1–4). Despite significant methodological advances in assessment of such target engagement among cells and tissues (5,6), preclinical testing often fails to fully capture human responses to drugs, leading to attrition in clinical trials because of insufficient efficacy or compromised safety (7,8). Protein kinases are a frequently targeted component of the druggable proteome, and the second largest target protein family for drug discovery (9–11). Kinases are involved in intracellular signal transduction in processes such as cellular growth, differentiation, and apoptosis in the course of normal cellular functions, and they play crucial roles in human diseases, notably in cancer (12,13). Key structural elements of the active sites are conserved across some 518 human kinases, and unwanted effects on off-target kinases is a common challenge in developing kinase inhibitors (14,15). In keeping with this notion, there is a broad repertoire of techniques available for kinase selectivity profiling many of which are based on inhibition of recombinant kinase activity or competition assays based on immobilized or labelled tracer molecules (16–17). It is instructive that drugs can maintain their target specificity despite being covalently anchored to a solid phase. Kinase target engagement can also be assessed in live cells using fluorescence or bioluminescence resonance energy transfer (FRET or BRET, respectively), provided both the drug molecule and its kinase targets can be suitably modified (18,19). Given the availability of exquisitely selective tracers such measurements can also provide spatial resolution both *in vitro* and *in vivo* using, e.g. fluorescence lifetime imaging microscopy (FLIM) (20,21). While the above-mentioned approaches are generally applied to one kinase at a time, there are also chemoproteomics approaches, where e.g. modified nucleotide analogs or activity-based probes are employed for a more complete understanding of kinase selectivity in lysed cells and tissues (22–24).

Target engagement can also be measured in live cells without or with only minimal prior modification of either interaction partner (25–35). One such technique is the cellular thermal shift assay (CETSA), which measures underivatized drug binding to endogenous proteins in live cells and tissues by monitoring thermal stabilization of target proteins (26–29). While significant progress has been made to allow CETSA measurements in tissues of treated animals, whole blood samples, and fine needle aspirates (30), such measurements can only be achieved with single cell resolution under special circumstances (30–33). Instead, localized measurement remain dependent on the use of radiolabeled drugs in low resolution imaging by positron emission tomography (PET) or the combined use of fluorescent tracers and fusion proteins to generate NanoBRET signals (6,34–35). These limitations illustrate the need for new approaches to measure physiologically relevant target engagement, and in particular for techniques that can provide both cellular and target protein resolution. Ideally such methods would allow probing for selective target protein binding in *ex vivo* pathological and normal human tissues to establish biomarkers of patient responses. Such methods have the potential to improve the outcome of clinical trials by eliminating sub-optimal candidate drugs already in preclinical research, and they could help select optimal targeted therapies in clinical routine (4,8). Recent advances in drug development include the preparation and screening of large collections of DNA-barcoded compounds, also known as DNA-encoded chemical libraries (DECLs), permitting single-pot screens of oligonucleotide-modified compounds (36–40). We hypothesized that it might be possible to use oligonucleotide-conjugated drugs as affinity reagents to visualize target engagement directly in human tissues. This is in analogy to our and others’ use of molecular genetics tools in antibody-directed rolling circle amplification (RCA) reactions, and for *in situ* proximity ligation assays (isPLA) (41–44).

**Figure 1.**
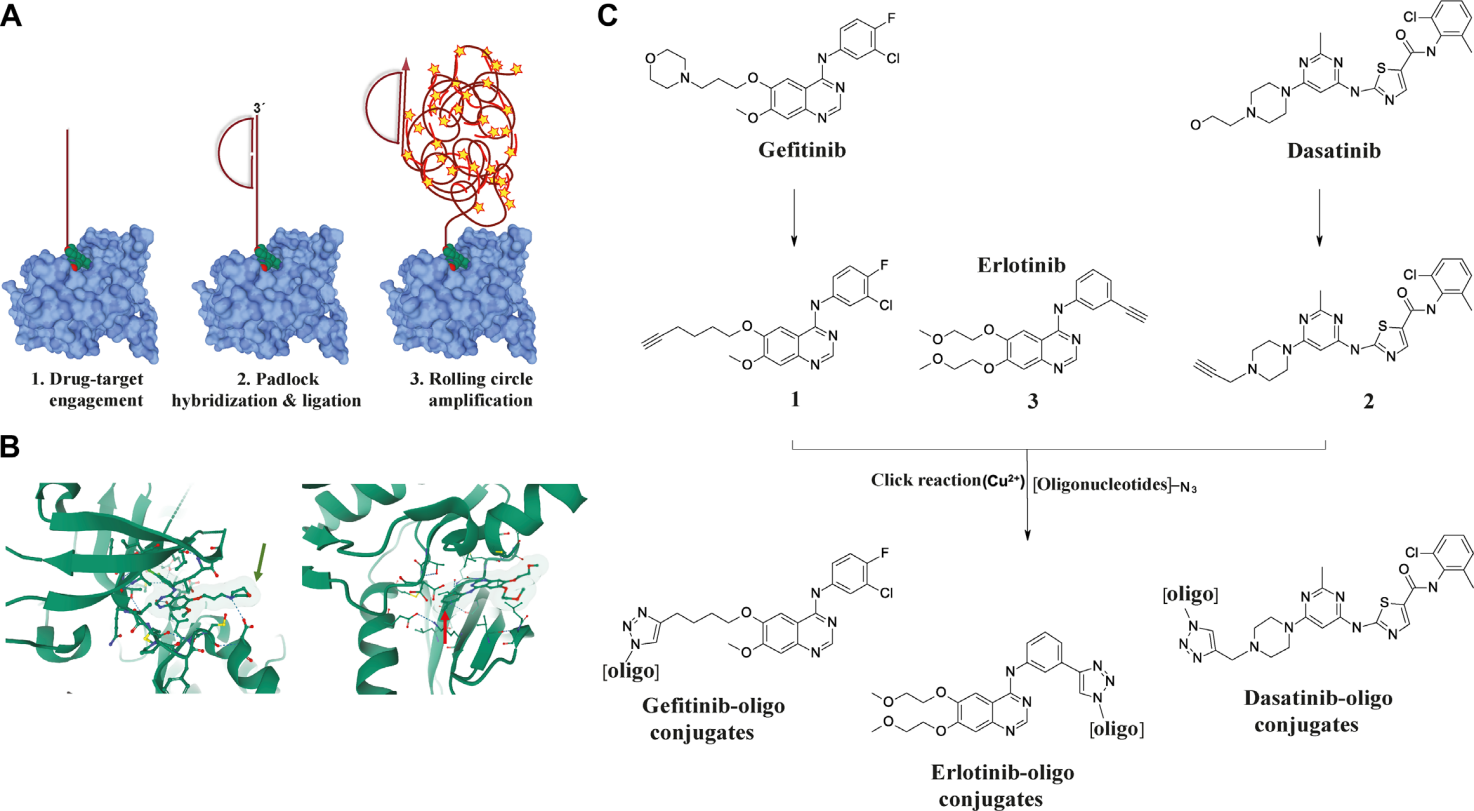
Principle of TEMA and design of EGFR-directed drug-oligonucleotide conjugates. (**A**) In TEMA, small molecule ligands conjugated to oligonucleotides bind their target proteins (1) in sample matrices such as protein arrays, fixed cells and tissue sections. After washing away excess ligand, any remaining bound conjugate is recognized (2) by padlock probes (54). These probes serve as substrates for ligation reactions and form oligonucleotide circles that template localized RCA reactions, the products of which are visualized using fluorescent oligonucleotide probes (3). The illustration of the EGFR kinase domain (light blue) is based on RSCB PDB (https://www.rcsb.org) entry 4WKQ (unpublished) using Mol* (55). (**B**) Illustration of binding by gefitinib (left) and erlotinib (right) in the active site of the EGFR kinase domain (53). While the morpholine of gefitinib protrudes out of the active site and represents a suitable exit vector for oligonucleotide conjugation (green arrow), the alkyne of erlotinib is buried in the active site such that conjugation causes steric hindrance (red arrow). The illustrations are based on RSCB PDB entries 4WKQ and 1M17 using Mol*. (**C**) Structures of gefitinib, a selective EGFR-targeted drug, and dasatinib, with a broader kinase inhibition profile (erlotinib was included as a control) (56). The morpholine in gefitinib, and the hydroxyl group of dasatinib were replaced with an alkyne in precursors 1 and 2 and click chemistry afforded conjugation to azide-modified oligonucleotides. The corresponding alkyne precursors of gefitinib (1) and dasatinib (2) were synthesized and used to generate gefitinib- and dasatinib-oligonucleotide conjugates, respectively, while the alkyne in erlotinib (3) allowed direct conjugation to provide erlotinib-oligonucleotide conjugates. Details on synthetic schemes and characterization of the molecules are available is Supplementary Information.

We describe herein this target engagement-mediated amplification (TEMA) method, and its application for investigating localized target and off-target engagement on protein arrays as well as in cells and tissues. We established TEMA using clinically approved tyrosine kinase inhibitors (TKIs), modified by covalently attaching oligonucleotides. These oligonucleotide-conjugated drugs were allowed to interact with their targets in model systems of increasing complexity, moving from protein arrays to cultured cell preparations and then sections from patient tissue samples. The localization of each physical interaction was visualized by amplification of circularized oligonucleotide probes via RCA, generating localized amplification products that can be visualized with hundreds of fluorophores for each bound drug molecule. The RCA reaction ensures detection over nonspecific fluorescence, and permits counting of individual reaction products (Figure 1a). In a variant of the technique, TEMA is combined with isPLA (proxTEMA) by applying the oligonucleotide-conjugated small molecules together with antibody-oligonucleotide conjugates to achieve molecular resolution in target engagement through isPLA. This form of the assay focuses the *in situ* analyses of drug binding to specific on- or off-target proteins in a sample. Besides revealing cellular target engagement in clinically relevant samples, oligonucleotide-conjugated drugs also significantly broaden the repertoire of affinity reagents for probing the presence and localization of proteins.

## MATERIALS AND METHODS

### Drug–oligonucleotide conjugation by click chemistry

Copper-catalyzed click reactions of drugs containing alkyne group and azide-modified oligonucleotide were carried out using Invitrogen's buffer kit (catalog no. C10276). The reaction stoichiometry was optimized using the alkyne-containing drug molecules in molar excess (5-, 10-, 15-, 25-, 50- or 125-fold) over oligonucleotides. The reaction mixture contained 2× concentrated Click-iT^®^ reaction buffer (component A), CuSO_4_ (II) (component B, 100-fold molar excess over drug) in 100 μM reaction catalyst tris[(1-benzyl-1*H*-1,2,3-triazol-4-yl)methyl]amine (TBTA, dissolved in DMSO). TBTA was added, followed immediately by the Click-iT^®^ reaction buffer additive 1 (component C; should be colorless, discard brown solutions). The reaction components were mixed and incubated for 3 min at room temperature (RT). The reaction mixture solution turned bright orange upon addition of the final reaction buffer component D (click-iT^®^ reaction buffer additive 2 solution). The reactions were continued for an hour at RT and stopped by purification using Zeba™ Spin desalting columns (7K MWCO, Thermo Scientific). The oligonucleotide-conjugates were further purified using Slide-A-Lyzer Dialysis Cassette (3K MWCO, Thermo Scientific) over night at 4°C in phosphate buffered saline (PBS) to remove excess unreacted drug molecules. The conjugates were identified and pure probes were isolated through size exclusion chromatography using a Superdex 200 column in a HPLC system.

### LC–MS characterization

Starting materials (oligonucleotides and drugs), reaction component and conjugates were run in a BEH (Ethylene Bridged Hybrid Technology from Water) C18 1.7 μm 2 × 50 mm column with the following mobile phases: (A) 400 mM hexafluoro-2-propanol, 10 mM triethylamine acetate pH 7 in 10% methanol and (B) 400 mM hexafluoro-2-propanol, 10 mM triethylamine acetate pH 7 in 90% methanol. The negative separation mode was applied in MS2 ‘Q1’, i.e. ‘Q1 full scan mode’ for full scan and MRM (multiple reaction monitoring) with a general span of 200 Da. Liquid chromatography was performed at a flow rate of 0.5 ml/min, with injection volume 7.5 μl, 80% A to 10% over 3 min in Waters XEVO TQ MS, coupled to an Acquity UPLC chromatographic instrument. All raw data were processed using the software Mass Lynx 4.1 (Waters Corp.).

### Surface plasmon resonance (SPR) biosensor analysis

A Biacore S51 SPR biosensor instrument (GE Healthcare) was used to characterize interactions between conjugates with known on- or off-target proteins. Human recombinant protein kinase ABL1 (ThermoFisher/Life Technologies) (20 μg/ml in 20 mM HEPES, pH 7.5) was immobilized directly on a CM5 biosensor chip surface by amine coupling, to a level of about 5000 RU. EGFR kinase domain L858R (ThermoFisher/Life Technologies) was immobilized directly on the biosensor chip surface by an amine-coupling procedure (32 μg/ml in 10 mM sodium acetate buffer, pH 4.0) resulting 11 000 RU. The running buffer contained: 50 mM Tris–HCl pH 7.4, 150 mM NaCl, 10 mM MgCl_2_, 1 mM MnCl_2_, 0.005% Tween-20. A 1:1 serial dilution of drug conjugates was injected over immobilized ABL1 at concentrations ranging from 0.06 to 2 μM. All experiments were performed at a flow rate of 30 μl/min and at 25°C and data was analyzed using Biacore S51 Evaluation software (GE Healthcare).

### Human protein microarray experiments

ProtoArray^®^ v5.1 (PAH05251020, ThermoFisher, Waltham, MA, USA), containing replicates of >9000 full length human proteins for a total 23 232 spots per array (Supporting File 1). The proteins were expressed as N-terminal glutathione *S*-transferase (GST) fusions, expressed in using the Bac-to-Bac^®^ Baculovirus Expression System available from Invitrogen and affinity purified under native conditions to retain their proper conformation. Oligonucleotide detection probes labeled with the fluorophore FarRed with emission wavelength similar to Alexa Fluor^®^ 647 recommended for the reader, or antibodies conjugated with FITC, were used to detect RCA products and GST tag-specific anti-GST antibodies (DyLight^®^ 550). The arrays were scanned using the CapitalBio LuxScan HT24 at two different wavelengths: F635 (red) for the FarRed detection probe and F635 (532) for the FITC-labeled detection probe or for anti-GST-conjugated DayLight^®^ 550, serving to detect total protein amounts per spot. Data acquisition, alignment and image processing were performed using the GenePix^®^ Pro microarray (v6.1) software. Statistical analysis of protein array data was based on log-transformed intensities and statistical software ‘R’. The histogram was created using ‘ggplot2 package R’.

### Cell preparation for TEMA

The cells were fixed in 3.7% paraformaldehyde on ice for 20 min and washed twice with DEPC-treated PBS, before being permeabilized with Tris-buffered saline with detergent (50 mM Tris–HCl, pH 7.5, 150 mM NaCl with 0.02% Triton) for 20 min. The cells were then washed twice with DEPC-treated PBS before TEMA experiments. For flow TEMA experiments, K562 and U937 cells were removed from the media by centrifugation and cell pellets were vortexted and washed once in 1xPBS, then fixed with 1% paraformaldehyde. A431 cells were trypsinized (0.25% trypsin/EDTA from Gibco) and then washed once in 1× PBS. All cells were fixed in 1% formaldehyde solution (Sigma Aldrich) in 1× PBS for 10 min on ice at RT. Cells were next pelleted to remove the fixation solution and washed in PBS. Permeabilization was performed by vigorous vortexing in 2 ml ice cold methanol (Sigma-Aldrich) and incubated for 10 min at 4°C, followed by two washes with 1× PBS + 1% BSA (New England Biolabs, Boston, USA).

### Fresh frozen tissue specimen

Human fresh frozen, fully anonymized breast cancer tissue sections were obtained from the biobank at the unit for Clinical Pathology at Uppsala University Hospital, Sweden in accordance with Swedish biobank legislation (ethical approval is not needed for research on fully anonymized human tissue specimens according to the Swedish Ethical Review Act (2003:460)). Human normal frozen colon tissue from the Medical University of Graz, Austria were provided by the Biobank Graz with ethics approval of the project under the ethical commission number 23-015 ex 10/11, entitled ‘Molecular and cellular characterization of colorectal cancer’. The tissue sections were stored at –80°C until fixation. The tissue sections were removed from storage at –80°C and fixed in ice-cold 3.7% formaldehyde for 20 min, and then permeabilized with TBS–0.02% Triton for 20 min. After permeabilization, the sections were washed twice in DEPC-treated PBS and immediately applied for experiments.

### Tissue microarrays

Formalin-fixed paraffin embedded brain tumor tissue array (T175a) including information about pathology grade and with normal brain tissue as controls, 12 cases/24 cores tissue specimens, were purchased from US Biomax. The tissue sections were prepared by deparaffinization through immersion in xylene for 20 min and then rehydrated in an ethanol series of 100%, 95% and 70% for 5 min each. Antigens were retrieved by pressure-cooking in Na-citrate buffer at pH 6.0 (10 mM sodium citrate, 0.05% Tween, 1× DAKO buffer diluted in water), washed with PBS and permeabilized with TBS–0.02% Triton for 20 min. After permeabilization, the tissue specimens were washed twice in DEPC-treated PBS and applied for in situ staining.

### TEMA experiments

All samples were incubated overnight at 4°C with blocking buffer containing 1 mg/ml BSA, 0.1 mg/ml salmon sperm DNA, 0.05% Tween-20 in 1× TBS. Samples were quickly washed once with purified PBS and immediately incubated with drug probes (Supplementary Figure S3; probes 1a or 2a or 3a) and if required also with antibody probes for 60 min in PBS at 37°C, except for experiments where protein arrays were incubated with drug probes at RT. After incubation, samples were washed with TBS-Tween 20 (0.001%) for 1 min and a quick wash with hybridization and ligation buffer. Hybridization and ligation were performed with ligation buffer for 30 min at 37°C. The ligation buffer contained 10 mg/ml BSA, 10× T4 ligase buffer, 1mM ATP, 250 mM NaCl, 0.001% Tween20, 125 nM padlock probe oligonucleotides (5’ phosphorylated PadlockP1-3, Supplementary Table S1) and 0.05 U/μl T4 DNA ligase in ddH_2_O. Next, samples were washed with 1× TBS for 2 × 2 min and again quickly washed with RCA buffer, followed by RCA with 0.5 unit/μl Phi-29 DNA polymerase (Fermentas) in RCA buffer (1× Phi-29 DNA polymerase-buffer (Fermentas), 250 nM detection oligonucleotides (Detection tagD1, Supplementary Table S1), 7.5 nM polyA (Sigma-Aldrich), 0.25 μg/μl BSA, 0.25 mM dNTP (Thermo Scientific), 0.001% Tween in ddH_2_O) incubated for 90 min at 37°C. The samples were washed twice with TBS–Tween 20 (0.05%) for 5 min, followed 1× TBS 2 × 5 min and quickly washed with purified water for 1 min at RT. Cells and tissues were stained with Alexa Fluor^®^488 phalloidin (1:50 in PBS- 0.01% BSA) and Hoechst dye (1:1000 in PBS–0.01% BSA) both for 10 min at RT. For protein array experiments goat anti-GST antibodies (DayLight^®^ 550) were diluted at 1:10 000 in PBS–0.1% BSA and incubated for 60 min at RT.

### TEMA with flow cytometry readout

1 × 10^6^ cells were incubated for 45 min at 37°C in the Odyssey^®^ Blocking Buffer in TBS containing 0.1% sodium azide (LI-COR Bioscience) after permeabilization and incubation. After centrifugation the blocking agent was decanted and the drug and antibody probes, diluted in PBS, were incubated with the cells for 90 min at 37°C. The cells were next washed with 1× TBS with 0.001% Tween 20 (Sigma-Aldrich), and incubated with 0.05 U/μl T4 DNA ligase in ligation buffer for the ligation step, followed by RCA. After one wash with TBS-0.01% Tween 20 the cells were placed in PBS and examined in a BD LSRII or BD Fortessa flow cytometer (BD Bioscience) using the PE-Texas Red filter. The gating in the plots shows the fluorescence of the samples. Gating was performed by placing a gate around detected cells compared to a negative control incubated with the same azide-modified oligonucleotide without a conjugated drug molecule, using forward and side scatters. The positive events were detected using an antibody probe and gated cells were counted. The BD FACSDiva software version 8.0 (BD Bioscience) was used for data analysis.

### ProxTEMA

All samples were incubated overnight at 4°C with blocking buffer containing 1 mg/ml BSA, 0.1 mg/ml salmon sperm DNA, 0.5% Tween 20 and 1× Tris-buffered saline. The samples were washed with PBS for 1 min at RT and for the regular *in situ* PLA experiment conjugated anti-EGFR, anti-BCR and anti-ABL antibodies probes were diluted 1:100 in Duolink antibody diluent buffer (Sigma-Aldrich) and incubated for 1 h at 37°C. For ProxTEMA, in a first step only the antibody probes (oligonucleotide-conjugated anti-EGFR, anti-BCR or anti-ABL), diluted 1:100 in Duolink antibody diluent buffer (Sigma-Aldrich), were incubated with the samples for 60 min at 37°C. After washes with TBS-Tween (0.01%) for 2 × 5 min, 2.5–10 nM oligonucleotide-conjugated drug probes (Supplementary Figure S3; probes 1b or 2b or 3b) were added and incubated for 90 min at 37°C in PBS, followed by washes with TBS-Tween (0.01%) for 2 × 2 min and then with ligation buffer for 1 min. Hybridization and ligation to generate circular reporter oligonucleotide strands were performed for 30 min at 37°C in ligation buffer containing 10 mg/ml BSA, 10× T4 DNA ligase buffer, 1 mM ATP, 250 mM NaCl, 0.05% Tween 20, 125 nM each of two phosphorylated oligonucleotides and 0.05 U/μl T4 DNA ligase in ddH_2_O. The two oligonucleotides can be ligated to form an oligonucleotide circle, templated by the oligonucleotides conjugated to pairs of drug and antibody probes having bound in proximity (Figure 5A and Supplementary Table S1). Samples were then washed with 1× TBS for 2 × 2 min and for one min in RCA buffer, followed by RCA for 90 min at 37°C with 0.5 unit/μl Phi-29 DNA polymerase (Fermentas) in RCA buffer. The samples were washed with TBS-Tween 20 (0.01%) for 2 × 5 min, in 1× TBS 2 × 5 min and quickly washed with purified water for 1 min at RT, protected from light.

### Image acquisition and analysis

Images were acquired using an ImageXpress Micro automatic plate scanning microscope from Molecular Devices and an epifluorescence microscope (Zeiss Axioplan2 image station and Zeiss AxioCam camera MRm). For analysis in 96-well plates nine 20x magnification images were collected per well as low-resolution overviews of the nine images in a 3 × 3 tile format using the MetaXpress software. CellProfiler software was used to analyze and quantify the images. Maximal intensity projection of the z-stacks was acquired using the CellProfiler cell image analysis software.

## RESULTS

### Design and synthesis of TEMA probes

We generated oligonucleotide-conjugated small molecule probes to explore the TEMA approach. For this purpose, we focused on tyrosine receptor kinases for which there are multiple approved drugs. This choice was based on several criteria: (i) availability of x-ray crystal structures of receptor-drug complexes; (ii) a good understanding of structure–activity relationships; (iii) literature precedence for conjugated drug analogs with retained binding to the primary targets, including examples with dissociation rates (*k*_off_) on the scale of hours (45–47), such that extensive washes can be employed as customary in immunofluorescence-based assays; (iv) literature data on kinase selectivity; (v) in-house access to relevant clinical material and (vi) availability of affinity reagents and prior experience in our labs of generating similar tool compounds. We also aimed to include compounds with different selectivity profiles, such that observations with TEMA could be cross-validated against reference data.

Given these considerations, we based our efforts on the specific epidermal growth factor receptor (EGFR) inhibitor gefitinib and the more promiscuous dasatinib, both of which have previously been modified at an exit vector protruding out of the active site of the target kinase (48,49). Especially dasatinib dissociates slowly from its primary targets, with half-lives on the order of several hours. We utilized this information to introduce an alkyne moiety in parts of these molecules not involved in kinase interactions (Figure 1B). Purified alkyne-containing drug analogs were further reacted with azide-labeled oligonucleotides via click chemistry to form triazole bonds between oligonucleotides and drugs (Supplementary Figure S1) (50–52). Given the natural presence of an alkyne in erlotinib, a broader-spectrum EGFR drug, we included this as a negative control since direct conjugation to its alkyne is expected to hinder active site binding in kinases (53). The resulting drug-oligonucleotide conjugates were purified and quality checked prior to application in biological assays (Supplementary Figures S2, S3 and Supplementary Table S2). We used surface plasmon resonance (SPR) biosensor analysis to investigate the binding of the conjugates to the target protein. The dasatinib probe interacted strongly with the on-target ABL1 kinase compared to the geftinib probe, and the dasatinib probe showed a slow dissociation rate for its target ABL1 kinase, negative control experiments showed no interactions for the azide-labeled oligonucleotides (Supplementary Figure S4). We observed moderate binding of the dasatinib and gefitinib probes with the EGFR L858R kinase domain at higher nanomolar probe concentrations, but only a very weak interaction was seen for the inactive erlotinib probe, which was used as negative control drug probe. These experiments confirmed that the drug-oligonucleotide conjugates had the expected interaction profiles for the selected target.

### Application of TEMA on protein microarrays

**Figure 2.**
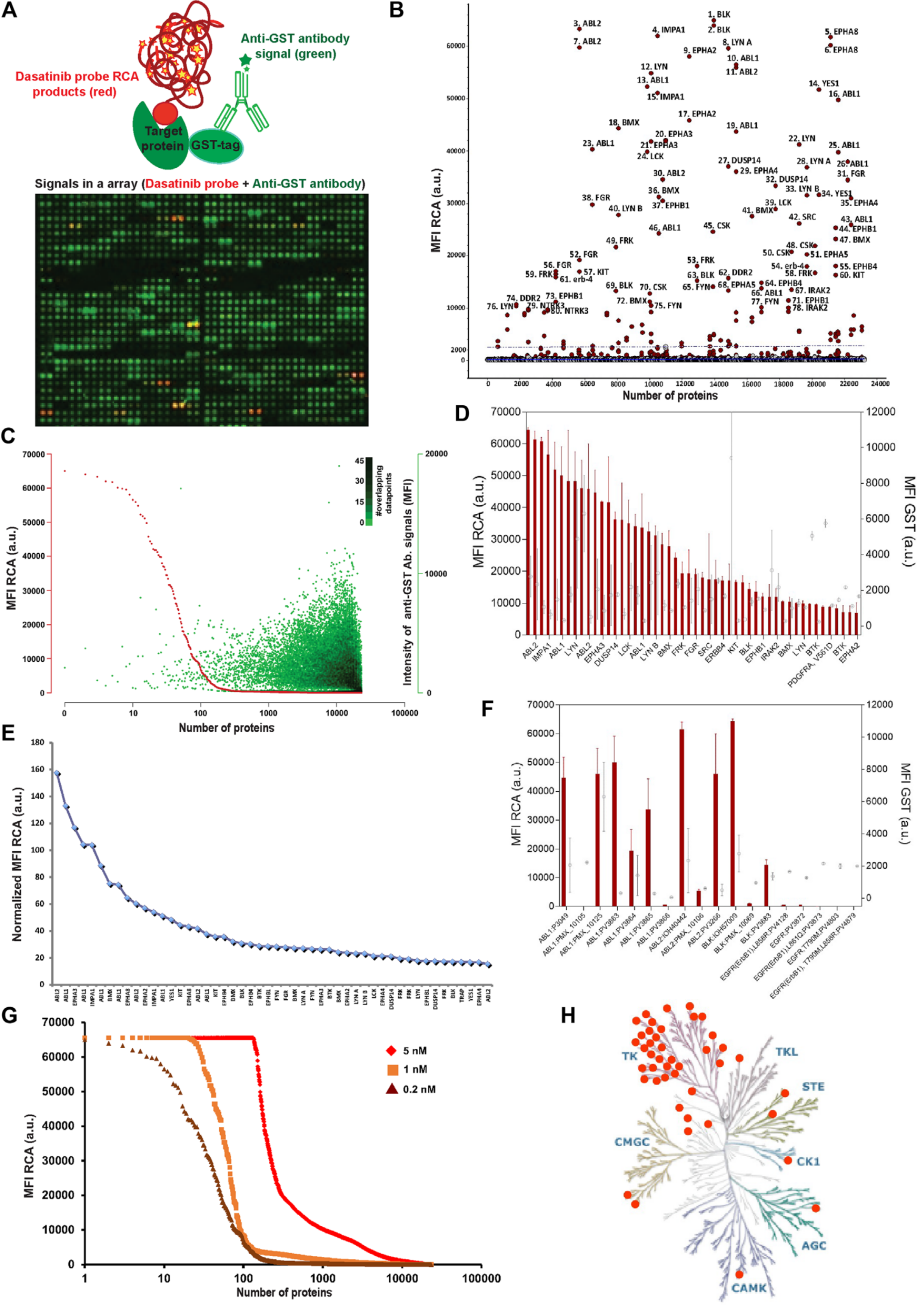
Array-based profiling of kinome specificity using TEMA. (**A**) Fluorescence signals from RCA products generated through binding of the dasatinib-oligonucleotide conjugates at 0.2 nM concentration to human proteins spotted in a microarray. Detection is achieved using a complementary oligonucleotide labeled with FarRed (red), while a Daylight 550-labeled antibody (green) directed against the GST tag was used to control for the variable amounts of individual spotted protein. (**B**) Proteins identified by TEMA using the dasatinib probe at 0.2 nM. The results illustrate signals from the 80 proteins yielding the highest signals (>10 000 a.u.), almost all of them kinases as identified in the hits list (Supplementary Table S3 and Supporting Data File 1). (**C**) The median signals for the dasatinib probe (red) added at 0.2 nM was plotted on a log scale and in logarithmic order of decreasing intensity for the 9,000 proteins with replicates helping to assess reproducibility—in total 23 234 human protein spots. The signals for antibody-mediated detection of the GST tags of the same spotted proteins are plotted in green (Supplementary Figure S6). (**D**), MFI signals for the top 25 dasatinib hits (red bars; left y-axis) and the corresponding signals for GST (○; right y-axis)—error bars represent the difference between two technical replicates. (**E**) Signals for the dasatinib probe at 0.2 nM binding to duplicate arrayed proteins as identified by TEMA (Supplementary Figure S5), corrected for the amounts of each protein as determined by normalization of anti-GST antibody signals. The results illustrate signals for the 50 proteins yielding the highest values, almost all of them kinases as identified in the hits list (Supplementary Table S3 and Supporting Data Files). (**F**) MFI signals representing the TEMA and GST responses of the dasatinib probe for multiple variants of the same protein (same symbols and experimental conditions as in B). (**G**) Investigation of the dasatinib target coverage by TEMA as a function of the concentration of the dasatinib conjugate – 0.2 nM (dark red line), 1 nM (orange line) and 5 nM (red line). (**H**) Kinome dendrogram based on the observed binding profiles of the dasatinib probe at 0.2 nM in arrays using TEMA with a cut-off of 2000 MFI. The human kinome map dendrogram was adapted with permission from Cell Signaling Technology (www.cellsignal.com).

Microarrays of displayed recombinant proteins represents a convenient format for fluorescent readout of discrete interactions, as previously demonstrated for oligonucleotide-conjugated antibodies following signal amplification via RCA (27–29). Current literature include applications that also require functionality of the displayed proteins, such as their interactions with small molecules and the identification of novel kinase substrates (57–59). All proteins in the commercially available ProtoArray® Human Protein MicroArray (Thermo Fisher Scientific) have been purified and arrayed under native conditions to allow such studies. We adopted this format for investigation of the binding profiles of the dasatinib-oligonucleotide conjugate. Fluorophore-labeled drugs have previously been used to measure binding in protein arrays (60). The oligonucleotide-conjugated constructed allowed for locally amplified detection via RCA. Circularizing oligonucleotides (padlock probes) were designed with 5′ and 3′ ends complementary to adjacent segments of the oligonucleotides conjugated to the drug molecules. Once converted to oligonucleotide circles by ligation, the probes were replicated through localized RCA, primed by the drug-conjugated oligonucleotides, and visualized using fluorescence-labeled hybridization probes to the repeated sequence of the RCA products (61,62). RCA offers a signal enhancement of several hundredfold over singly fluorophore labeled compounds, permitting visualization of even single bound drug probes (Figure 2A).

Using this approach the dasatinib–oligonucleotide conjugate was applied to planar arrays of >9000 human proteins spotted in two technical replicates (Supplementary Figure S5). The displayed proteins contained a glutathione S-transferase (GST) tag, allowing for control of levels of displayed protein using a fluorescence-labeled antibody that specifically recognizes GST with high affinity (Supplementary Figure S6). Following incubation, washing, ligation, RCA amplification, and hybridization we recorded median fluorescence intensities (MFIs) for the spotted proteins, ranging from 25 to 64 992 arbitrary units for the dasatinib-oligonucleotide conjugate (Figure 2C). We observed prominent TEMA responses for a subset of ∼50 proteins (Figure 2B, C). A significant portion of those were relevant tyrosine kinases, with the strongest signals observed for known dasatinib targets such as Abl1, Abl2, Blk, Yes, Lyn, Src, PDGFRA and members of the ephrin receptor subfamily (Figure 2B, C). The hit list also contained a smaller number of protein phosphatases, with inosine monophosphatase 1 (IMPA1) and dual-specificity protein phosphatase 14 (DUSP14) at the top of this list (Figure 2B, D, E and Supporting Data File 1).

The observed kinase binding profile using TEMA was in broad agreement with published selectivity data for dasatinib (Figure 2H, Supplementary Figure S8 and Supplementary Table S3) (56), suggesting a significant portion of the arrayed proteins are functional. However, a small number of known dasatinib targets were missed (e.g. CSF1R, EPHB6, GAK and MAP2K5) using a probe concentration of 0.2 nM. This prompted follow-up experiments at concentrations ranging between 0.2 and 5 nM (Figure 2G), but these four kinases remained undetectable when tested with the dasatinib probe at 1 nM, and the experiment at 5 nM concentration did not yield a specific set of targets due to elevated background binding. The anticipated interactions with CSF1R and EPHB6 in the protein arrays, with reported sub-nM potencies (63), were thus missed across the TEMA experiments, while lack of signals for the low nM binders GAK and MAP2K5 could potentially be explained by the application of insufficient probe concentrations.

We undertook several experiments to understand the reason for the missed targets. First, we looked at the variability between technical replicates versus differences in response between different forms of the same kinase (included as separate products on the microarray). This is exemplified for top kinase hits in Figure 2B, D, where strong signals (>10 000 a.u.) were observed for five out of seven spotted variants of Abl1, while two of the spotted protein variants resulted in background signals only (this was true for both replicates of the two proteins). Similarly, for Abl2 and Blk two out of three variants showed positive responses (see Supplementary Figure S7 for additional comparisons across the Ephrin receptor tyrosine kinases). These observations were not due to variation in amounts of spotted proteins, as significant GST signal were recorded for several of the inactive protein variants. Since EPHB6, GAK and MAP2K5 were only present once each in the microarray, and CSF1R twice, we conclude that the lack of observed binding for the dasatinib probe may be because these proteins were non-functional.

Of particular note in this study was the lack of responses for all included forms of the EGFR receptor (Figure 2F), demonstrating insufficient affinity of the dasatinib-oligonucleotide conjugate for retention on spotted EGFR variants during washes, possibly due to lack of functional proteins on the microarray. Reported affinities for dasatinib to different EGFR mutants range from 20 nM into the μM range (17,56,63). The application of the probe at 0.2 nM may therefore have been insufficient to allow retention of drug conjugates during the TEMA experimental procedure. Although a larger fraction of the proteins in the array exhibited detectable signals in follow-up experiments at 1 nM (Figure 2G), none corresponded to the spotted EGFR proteins. Experiments were also performed at 5 nM probe concentration, resulting in significantly higher background binding but none of the EGFR products gave signals above a 10 000 MFI threshold (Supporting Data File 1). We conclude that although the manufacturer does not guarantee correct folding of proteins displayed on the arrays, the TEMA results show high intra- and inter-assay reproducibility and excellent agreement with results using other approaches for *in vitro* profiling of kinase inhibition. We deem it less likely, but cannot exclude, that the oligonucleotide-conjugation of dasatinib selectively inhibits binding to EGFR, while preserving the affinity for other target proteins.

### *In situ* TEMA reveals spatially resolved drug binding in cells and tissues

**Figure 3.**
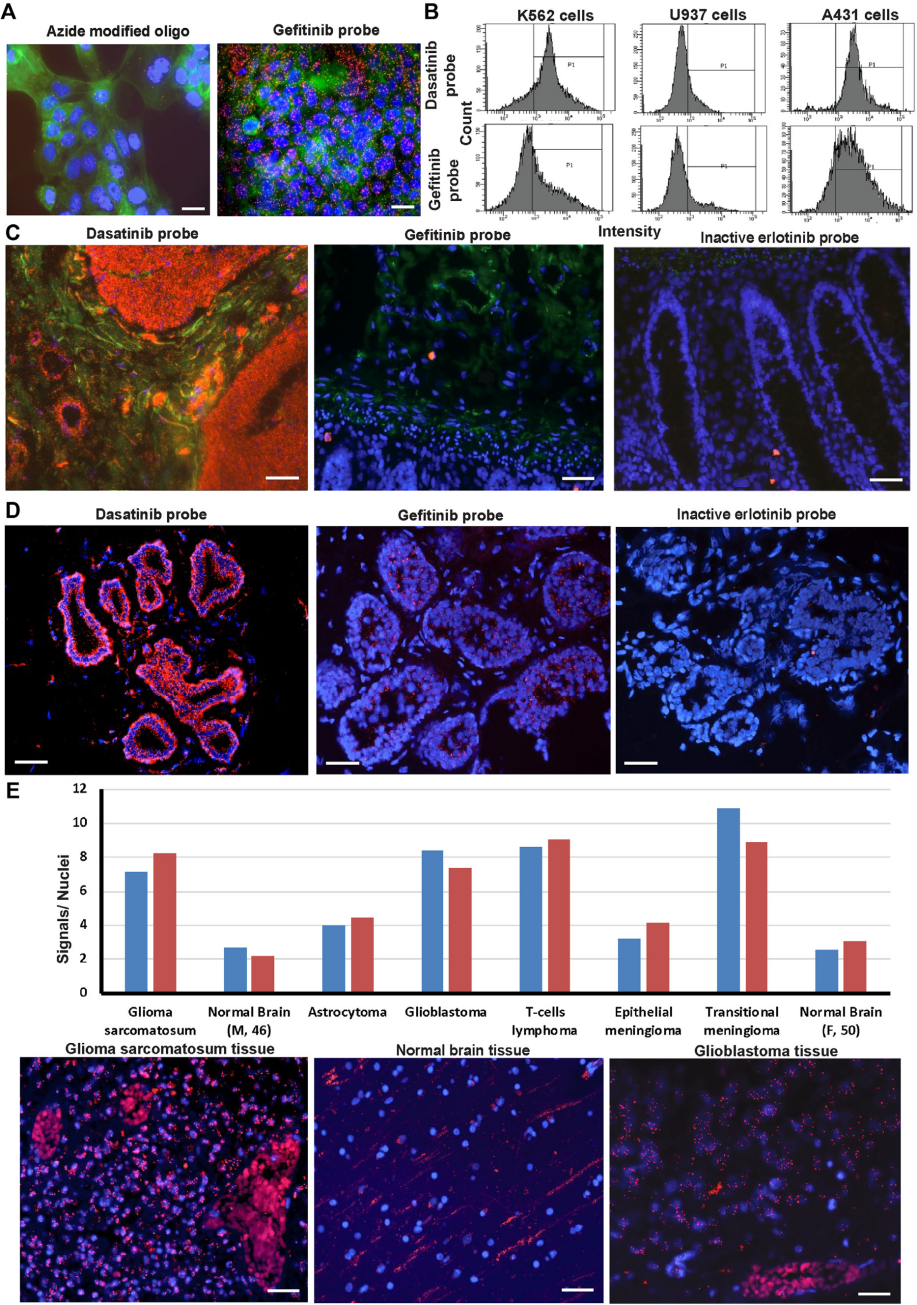
Localization of drug binding in tissues by TEMA. (**A**) Binding of gefitinib probe (at 5 nM; red dots) in the cancer cell line A431, expressing high levels of EGFR, and the azide modified oligonucleotides without conjugated drug molecules, used as a negative control. Cytoplasms and nuclei were stained with phalloidin (green) and DAPI (blue), respectively. The images were acquired by fluorescence microscopy. Scale bars represent 20 μm. (**B**) Flow cytometry analysis by TEMA for quantitative comparisons of target engagement by kinase inhibitor probes (5 nM) in K562 cells, overexpressing the ABL kinase as a fusion protein, and A431 cells, expressing EGFR transcripts at higher levels compared to U937 cells having low/absent EGFR expression. (**C**) Comparison of binding by gefitinib and dasatinib probes at 1 nM in TEMA analyses of fresh-frozen normal human colon tissue sections. TEMA signals are seen in red, while nuclei in the tissues were stained blue using DAPI. (**D**) Investigation of fresh-frozen breast cancer tissue sections, previously scored as 3+ for HER2 protein staining by the HercepTest (Dako), indicating high expression of HER2. TEMA probes at 1 nM signals are seen in red. (**E**) Binding by gefitinib probes at 5 nM added to formalin-fixed paraffin-embedded brain cancer tissue sections and normal brain tissue in a commercial tissue microarray. The numbers of RCA products, representing TEMA gefitinib signals, were quantified per nuclei using CellProfiler software. The pairs of bars in distinct colors show duplicate observation for on average >2000 cells in each sample type. RCA products are seen in red. Scale bars in panels (C)–(E) are 50 μm.

For sufficiently specific probes, binding experiments can be extended to increasingly relevant model systems, i.e. going from isolated proteins and functional arrays to whole cells and tissues (20,21). The latter aspects are of particular interest as they allow analyses of patient material that are otherwise notoriously difficult to achieve. Insight into the distribution of target binding drugs in cells and tissues can confer valuable information about the clinical potential of drug candidates. As the oligonucleotide-conjugated variants of the TKIs retained strong and specific binding for their target proteins, we therefore sought to further examine this binding in situ in cells and tissues. We observed that levels of TEMA signals for five cell lines correlated well to their EGFR RNA expression as documented in the Human Protein Atlas (HPA) RNA-seq cell line dataset (www.proteinatlas.org); A431: 337.7, HaCAT: 229.9, SK-BR-3: 47.8, MCF-7: 3.1 pTPM (transcripts per million) and U937, very low expression of kinases and EGFR (Figure 3A, B). The results were also in agreement with protein expression analyses by immunoblotting using two different EGFR-specific antibodies (Supplementary Figure S9). We note that the gefitinib probe RCA signals appears to be distributed to the nuclei in A431 and SKBR3 cells (Figure 3A and Supplementary Figure S11b).

HaCAT cells, grown in microtiter wells, were fixed and treated with variable concentrations of dasatinib probes, followed by detection via RCA. The numbers of RCA signals per cell were recorded by automated high-throughput image acquisition microscopy and image analysis using CellProfiler software (www.cellprofiler.org). Dasatinib probes at 100 pM generated strong, easily detectable fluorescent RCA products, and signals reached saturated levels at 10 nM concentrations (Supplementary Figure S10). The gefitinib probe at 5 nM generated strong localized fluorescence signals, representing interactions with target molecules in A431 cells that express high levels of the targeted EGFR protein (Figure 3A). The oligonucleotide-conjugated form of the narrow-spectrum clinical kinase inhibitor gefitinib generated significantly more signals in A431 cells, overexpressing the primary target EGFR, compared to K562 cells and the human macrophage cell line U937, where only few signals were observed (Figure 3B). These results are consistent with the HPA RNA-seq cell line dataset for K562: 0.0 and U937: 0.1 TPM, reflecting no or only very low expression of mRNA for the EGFR receptor in those cell lines. In contrast, the broad-spectrum clinical kinase inhibitor dasatinib exhibited higher signal intensity in the cancer cell line K562 overexpressing ABL along with other known target proteins for this drug (Figure 3B). Additionally, we compared the distribution of binding by the dasatinib and gefitinib probes in SK-BR-3 cells by labeling their RCA products with two distinct fluorophores, revealing a preferentially perinuclear staining by the gefitinib probe but broader signal distribution by the dasatinib probe (Supplementary Figure S11). The experiment illustrates the possibility to explore drug binding at subcellular resolution by TEMA.

We also used the drug-oligonucleotide conjugates to investigate the localization of drug binding in formalin-fixed healthy human colon tissue, expressing quite low EGFR levels, and in fresh-frozen breast cancer tissue. Strong dasatinib binding was observed in parts of the colon tissue, while gefitinib produced weaker staining (Figure 3C). In breast cancer tissue the gefitinib probe specifically localized in tumor-containing parts of the sections, thus revealing the site of drug binding in this clinical material (Figure 3D). An inactive probe, based on the kinase inhibitor erlotinib, was prepared by conjugating oligonucleotide to a region of the molecule known to engage in protein kinase targets binding. As expected, this nonfunctional probe resulted in weak to nonexistent staining in breast cancer tissue (Figure 3D). The gefitinib probe gave stronger detection signals in formalin fixed gliosarcoma and glioblastoma tissue microarray samples compared to other investigated cancer tissues, while normal brain tissue exhibited only very few signals (Figure 3E and Supplementary Figure S12). The TEMA findings lend support to earlier studies suggesting that gefitinib-like model kinase inhibitors may be attractive candidate molecules to treat brain cancer patients (64,65). We conclude that TEMA can generate *ex vivo* data revealing the distribution of drugs at cellular and subcellular resolution, as a basis for evaluating their suitability as clinical drugs.

### Competition experiments with unmodified drugs

**Figure 4.**
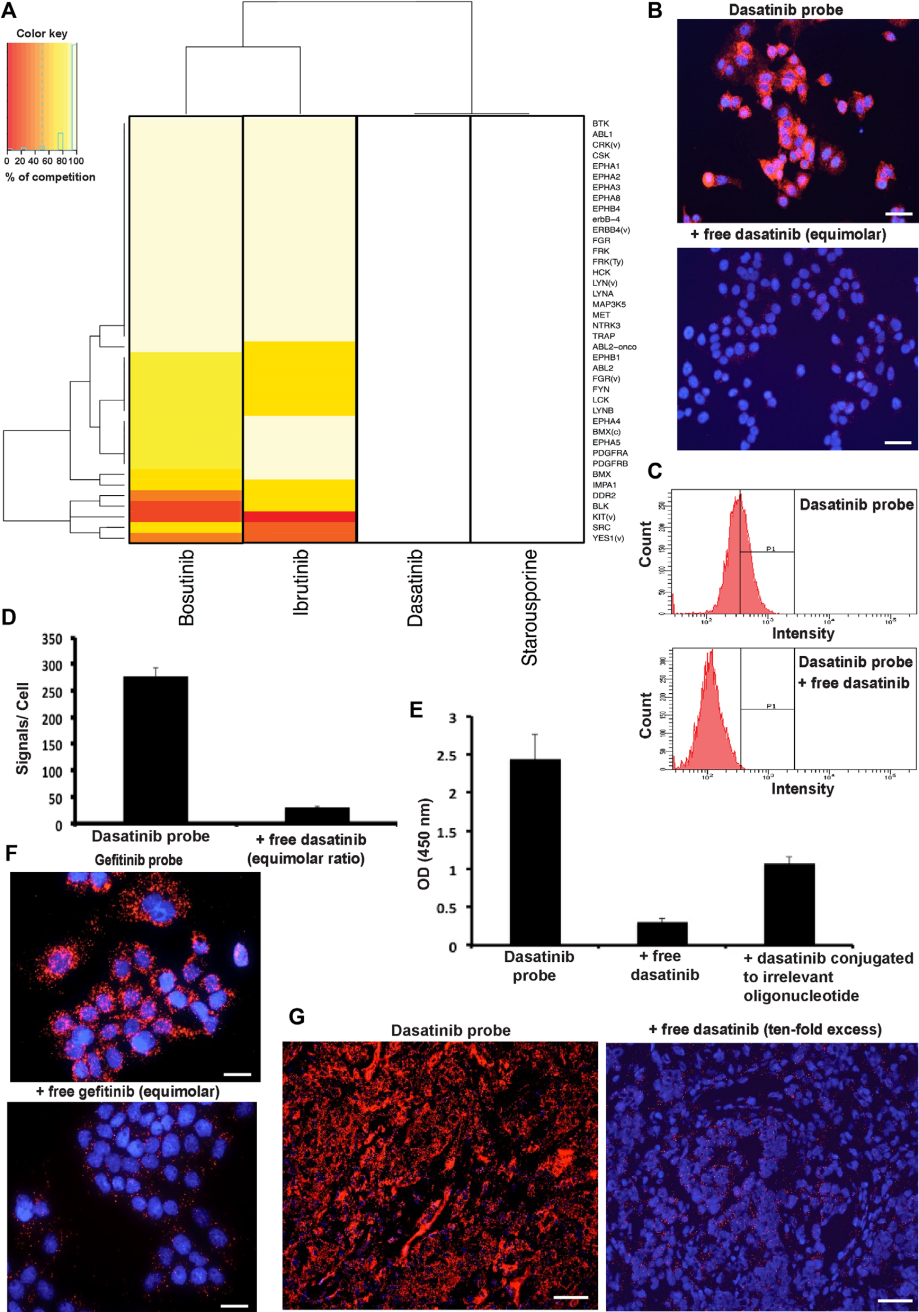
Competitive TEMA on arrays, in cells and in tissue sections. (**A**) Heat map illustrating competition between the dasatinib probe and four underivatized drugs on protein arrays. The dasatinib probe was added at 0.5 nM, alone or in competition with equimolar amounts of the unmodified kinase inhibitors dasatinib, staurosporine, bosutinib or ibrutinib. Dasatinib and staurosporine both completely inhibited binding by the dasatinib probe, while bosutinib and ibrutinib mainly inhibited binding to target proteins that are known to be shared with the dasatinib probe. The plot was generated using an in-house script developed in ‘R’. Data represent means from two independents experiments). (**B**) The dasatinib probe was applied at 1 nM to fixed MCF7 breast adenocarcinoma cells in the absence or presence of equimolar amounts of the unconjugated dasatinib drug, demonstrating strong diminution of numbers of RCA products by this competition with the unmodified original compound. (**C**) Flow cytometry readout of fixed A431 cells stained with a dasatinib probe applied at 10 nM alone or in competition with a 10-fold molar excess of unmodified dasatinib. The signals on the Y-axes reflect numbers of positive cells, while the fluorescence intensity per cell is indicated along the X-axes. Signals were recorded using a BD LSRII flow cytometer (in other experiments a BD Fortessa was used with similar results) with PE-Texas Red filter and the data were processed using BD FACSDiva software version 8.0. (**D**) Competitive binding signals for the dasatinib probe at 1 nM in A431 squamous carcinoma cells by equimolar unlabeled dasatinib. The TEMA signals were quantified per nuclei using CellProfiler software and the bars show values for duplicate observation. (**E**) A plot of total absorbance per well by colorimetric readout in cells using a microplate reader at wavelength 450 nm and absorbance at 650 nm as a reference. The bars show mean values of quadruplicate measurements with standard deviation (±). (**F**) Competitive binding signals by the gefitinib probe at 5 nM in A431 cells in the presence or absence of the unmodified gefitinib drug. Scale bars, 50 μm. (**G**) Competitive binding by the dasatinib probe in fresh-frozen breast cancer tissue with or without competition by a 10-fold molar excess of unmodified dasatinib.

We explored the competitive mode for drug binding on arrays as well as in cells and tissues, demonstrating that signals could be reduced by competition both with unconjugated dasatinib drug molecules and with dasatinib molecules conjugated to oligonucleotides that themselves could not give rise to detection signals via RCA. We also performed competition experiments against a set of underivatized drugs with partly overlapping specificity to that of dasatinib. In analogy with other competitive binding formats (16,18–19,56), specific binding of TEMA probes to relevant kinases is expected to depend on the availability of active sites, where probes and drugs compete for binding. Thus, prior treatment with saturating concentrations of unmodified drugs or endogenous substrates should prevent probe binding. As expected, both dasatinib and the experimental compound staurosporine, having a broad kinase inhibition profile, completely blunted the RCA signal from the dasatinib probe when applied at equimolar concentrations (Figure 4A). Bosutinib and ibrutinib also competed with the dasatinib probe, but complete competition was restricted to kinase targets for which their respective activities overlap with those of dasatinib (15,16,32–34). Thus, significant responses were retained for BLK, KIT, DDR2, SRC, LCK, ABL1, ABL2, IMPA1, BMX, YES1, EPHB1 and PDGFRB also in the presence of equimolar concentrations of these inhibitors (see Supplementary Table S4 for a more complete comparison).

These experiments demonstrate that protein microarray-based TEMA allows qualitative assessment of specificity profiles of kinase drugs, while the variability in retained function across spotted proteins precludes a complete quantitative assessment. An important prerequisite for these studies is a sufficiently slow off-rate of derivatized drugs for their primary target proteins, such that the compounds are retained during the extensive washes in the protocol. Unmodified dasatinib decreased signals from the dasatinib probe in A431 cells by an order of magnitude when present at equimolar concentrations in the reaction, whereas signals as expected were decreased by around one half with dasatinib, conjugated to an irrelevant oligonucleotide of a similar size (Figure 4E and Supplementary Figure S13b). This illustrates that as expected free drug binds the targets more readily than the corresponding drug-oligonucleotide conjugates, which has a greatly increased molecular weight compared to the native drug. A ten-fold molar excess of the unmodified dasatinib drug molecules greatly reduced staining by the dasatinib-oligonucleotide conjugate in pathological tissue sections (Figure 4G). We also observed similar competitive inhibition of binding by the gefitinib probe at 5 nM concentration in A431 cells with equimolar unmodified gefitinib (Figure 4F). The experiments illustrate that binding by the oligonucleotide-conjugated compounds qualitatively reflects the properties of the unmodified drugs.

### Analysis of specific target engagement via isPLA (proxTEMA)

**Figure 5.**
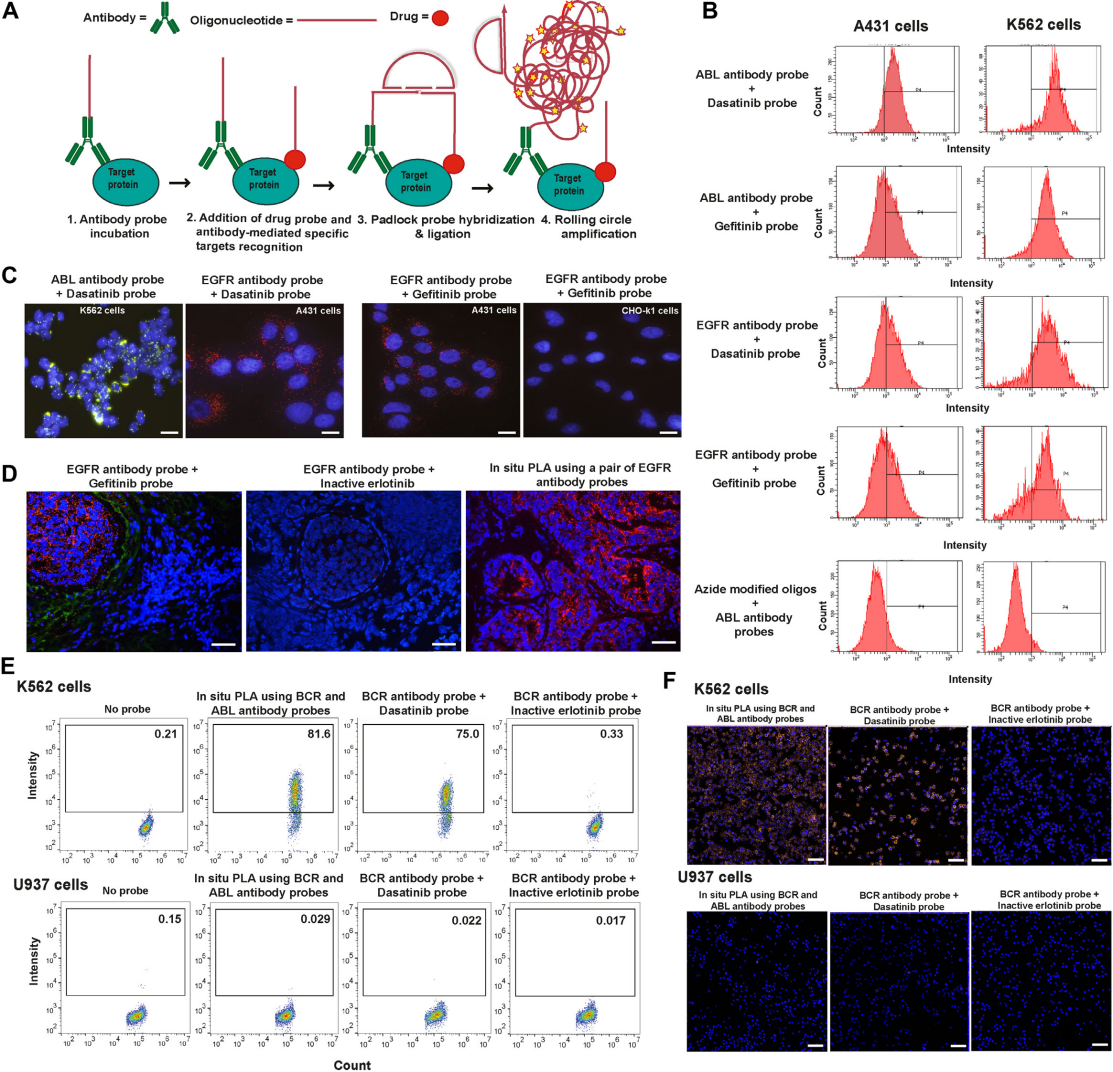
Schematic of proxTEMA and application in cell lines and fresh frozen tissues sections. (**A**) Using the proxTEMA approach drug molecules with conjugated oligonucleotides were combined with oligonucleotide-conjugated antibodies directed against a protein of interest. In cases where both reagents bound the same or nearby target molecules their attached oligonucleotides could template the formation of oligonucleotide circles by ligating pairs of added oligonucleotides. Once formed, the oligonucleotide circles were replicated in local RCA reactions, primed by the oligonucleotides conjugated to the antibodies. Successful detection depends on proximal binding by oligonucleotide conjugates of both drugs and antibodies. (**B**, **C**) Example of antibody-guided analysis of drug interactions with specific target proteins via proxTEMA by ABL or EGFR antibodies, combined with dasatinib or gefitinib probes (5 nM). (B) The antibody- and drug-probes were used in isPLA reactions, applied to the cell lines K562 and A431, both positive for ABL and EGFR, and the results were analyzed by flow cytometry. An assay where oligonucleotides having no conjugated drug molecules were combined with anti ABL antibody probes served as a negative control. (C) dasatinib (5 nM) was combined with anti-EGFR for isPLA analysis by microscopy of K562 cells (green signals) and with-ABL antibody probes for analysis of A431 cells (red signals). Similarly, gefitinib probes (5 nM) were applied with anti EGFR antibody probes for analysis of the A431 cells; positive for EGFR, and with CHO-K1 cells negative control for EGFR expression. Cell nuclei were counterstained using DAPI (blue) and images were acquired by fluorescence microscopy. Scale bars represents 20 μm. (**D**) Analysis of breast cancer sections by proxTEMA using anti-EGFR antibody probes together with 10 nM gefitinib probes or inactive erlotinib probes. Signals are shown in red. For comparison a pair of EGFR-specific oligonucleotide-conjugated antibody probes were combined for isPLA to serve as a positive control. The breast cancer tissue sections had been scored as 3+ with respect to HER2 staining using a HercepTest (Dako). The tissues were counterstained using DAPI (blue) and images were acquired by fluorescence microscopy. Scale bar represents 50 μm. (E, F) ProxTEMA was used for detection of the BCR-ABL fusion proteins by flow cytometry using an anti-BCR antibody-oligonucleotide conjugates together either with an anti ABL antibody-oligonucleotide conjugates, serving as a positive control, or with either 2.5 nM of dasatinib or the inactive erlotinib probe. The assays were performed in K562 cells, expressing the BCR-ABL fusion protein, and in U937 cells negative for this fusion protein. The experiment was repeated three times with similar results. K562 and U937 cells were counterstained using DAPI (blue) and images were acquired by fluorescence microscopy for in situ detection of BCR-ABL using an anti-BCR antibody-oligonucleotide conjugates together with 5 nM of drug probe. Scale bar represents 50 μm.

The TEMA technique described so far reveals all instances of drug binding in cells, tissues and protein arrays, whether to on- or off-targets. We decided to adapt the technique in order to focus on the characteristics of drug binding to particular target proteins in situ. For this purpose, we developed proxTEMA assays as a variant of the isPLA technique, previously mainly applied for antibody-based protein detection although with numerous variants (44), including an activity-dependent isPLA platform shown to be applicable for competitive measurements of drug binding (66,67). To the best of our knowledge, oligonucleotide-conjugated antibodies were here for the first time combined in isPLA with oligonucleotide-conjugated drug molecules applied as affinity reagents for potential target proteins of interest (Figure 5A). In this form of the assay, proximal binding by oligonucleotide conjugates of drugs and of protein-specific antibodies is required to give rise to the oligonucleotide circles that serve to template for RCA. This approach focuses the analysis of drug binding to specific on- or off-targets according to the antibody used (Figure 5A and Supplementary Figure S14d).

As a proof-of-concept, we confirmed binding of dasatinib and gefitinib probes to the ABL protein in fixed A431 and K562 cells in antibody-assisted analyses of drug interactions with specific target proteins via proxTEMA (Figure 5B, C). The dasatinib and gefitinib probes, binding endogenous ABL proteins, successfully generated proxTEMA fluorescent signals together with an anti ABL antibody probe. In contrast, azide-modified oligonucleotides without a conjugated drug failed to produce proxTEMA signals when combined with the anti ABL antibody probe, serving as a negative control (Figure 5B, C). We applied the proxTEMA approach by evaluating the specific binding of the gefitinib probe to the EGF receptor. The gefitinib probe was applied together with an oligonucleotide-conjugated anti-EGF receptor-specific antibody probe both in fixed A431 cells and in fresh-frozen breast cancer tissues scored as grade +3 HER2 positive (Figure 5C, D and Supplementary Figure S14a). The selectivity of the proxTEMA results were confirmed by analyses of EGFR negative CHO-K1 cells, and of normal human fresh-frozen colon tissue expressing very low EGFR levels as well as of breast cancer tissue section characterized as 0+ according to HER2 protein staining (Figure 5C, D and Supplementary Figure S14b, c). By contrast CHO-K1 cells and normal human fresh-frozen colon tissue failed to display significant signals, consistent with their lack of expression of EGFR (Figure 5C, Supplementary Figure S9 and S14b, c). We further demonstrated specific interactions by the dasatinib probe with the ABL kinase in BCR-ABL fusion protein positive K562 cells. We applied an oligonucleotide-conjugated anti-BCR antibody probe in combination either with anti-ABL kinase-specific antibody-oligonucleotide conjugate, or with the dasatinib probe as the pairs of affinity reagents, in both cases demonstrating the fusion proteins in K562 cells, with no signals in BCR-ABL fusion protein negative U937 cells (Figure 5E, F, Supplementary 14d). The dasatinib probe can bind the endogenous ABL proteins kinase domain while the structurally inactive erlotinib probe cannot. The inactive erlotinib probes having a low or no affinity for the ABL kinase, failed to produce proxTEMA signals when combined with the anti BCR antibody probes for detection of the BCR-ABL fusion proteins, providing a negative control for the analysis (Figure 5E, F). For positive control detection of BCR-ABL fusion proteins in K562 and U937 cells analyzed by a pair of BCR and ABL-specific oligonucleotide-conjugated antibody probes were combined for isPLA (Figure 5E, F). The results demonstrate that proxTEMA confers the unique ability to image drug binding to specific target proteins, directly in biological specimens.

## DISCUSSION

Information about target engagement by drugs in relevant tissues is crucially important in the drug discovery process, and also in choosing the optimal therapy among growing numbers of alternatives in the clinic. We show that TEMA can reveal both specific and cross-reactive target engagement of drugs and drug candidates by applying the compounds with attached oligonucleotide strands. The approach enables highly sensitive and reliable visualization of the localization of binding of drugs to thousands of arrayed human proteins, in searches for compounds with specificity for particular target proteins, and with minimal cross reactivity for undesired target molecules.

Another application of the TEMA technique allows high-content imaging of drug binding with digital quantification in cell preparations and tissue sections. We demonstrate that the assays successfully recapitulate binding characteristics of the unmodified drugs. The assays require only standard lab equipment like microscopes or flow cytometers, and they afford excellent sensitivity, reproducibility and a potential for multiplexing. TEMA can help establish selectivity profiles and risks of toxicity, either by attaching an oligonucleotide to the drug candidate, or by investigating competition by unmodified drugs to a oligonucleotide-conjugated drug molecule for binding to a specific target site. We applied this competitive assay format herein to establish that drugs conjugated to irrelevant oligonucleotides exhibit qualitatively similar abilities to compete for binding to those of the corresponding unmodified drug molecules. Besides competition with unmodified compounds, we also employed other controls such as the inactive erlotinib-oligonucleotide conjugate, and cells known to express or not express target proteins. Moreover, the protein array experiments provide abundant positive and negative controls for binding of the drug conjugates.

We note that the drug dasatinib, central to our experiments, has a long residence time. Further experiments will be needed to see how TEMA will perform against a broader range of drug targets and with reagents having faster off-rates. Nonetheless, we conclude that the oligonucleotide-drug conjugates investigated herein faithfully represent the properties of unmodified drugs albeit with some reduction of binding affinity compared to the unmodified compounds. These findings were corroborated by competition experiments using kinase inhibitors with either overlapping or non-overlapping target specificities, and translated to pathology tissue sections.

Finally, by combining oligonucleotide-conjugated drugs with similarly modified antibodies, the *in situ* drug binding assays can be focused on target proteins of particular interest, using an isPLA mechanism. The isPLA approach also provides an opportunity to minimize washes for detection of drug-oligonucleotide conjugates with relatively short dissociation times as discussed in the legend for Supplementary Figure S14. Assays similar to those described herein may prove suitable for analyses of e.g. broadly accessible fine needle biopsy material from tumor patients to establish the suitability of a given drug regime before therapy selection and to evaluate the emergence of resistance mutations. In summary, TEMA represents a new approach to demonstrate target engagement by drugs in relevant biological material, suitable for application in the drug discovery process and with a potential for predicting patient responses to drugs.

## DATA AVAILABILITY

The authors declare that the main data supporting the findings of this study are available within the article and its supporting files. Data and source codes have been deposited to FigShare, https://doi.org/10.6084/m9.figshare.21107641.v1.

The microarray data has been deposited in ArrayExpress under accession E-MTAB-12192. R and R-studio have been used to visualize data by allowing the software to plot individual data points along the X and Y axes. Code is available from the corresponding author upon reasonable request.

## Supplementary Material

gkac842_Supplemental_Files
