# Supplementary material for: Monitoring drug–target interactions through target engagement-mediated amplification on arrays and in situ
Source: Nucleic Acids Res. 2022 Oct 3;50(22):e129. doi: 10.1093/nar/gkac842 (PMC9825164; doi:10.1093/nar/gkac842)
Supplement: gkac842_Supplemental_Files [file gkac842_supplemental_files.zip › Supplementary Information.pdf]

Supplementary Results and Information

Title: Monitoring Drug-Target Interactions through Target Engagement-Mediated Amplification on Arrays and *in situ*

Rasel A. Al-Amin[1,*], Lars Johansson[2], Eldar Abdurakhmanov[3], Nils Landegren[4,5], Liza Löf[1], Linda Arngården[5], Andries Blokzijl[1], Richard Svensson[6], Maria Hammond[1], Peter Lönn[1], Johannes Haybaeck[7,8], Masood Kamali-Moghaddam[1], Annika Jenmalm Jensen[2], Helena Danielson[3], Per Artursson[6], Thomas Lundbäck[2], Ulf Landegren[1,*]

[1]Department of Immunology, Genetics and Pathology, Science for Life Laboratory, Uppsala University, Sweden.
[2]Department of Medical Biochemistry and Biophysics, Chemical Biology Consortium Sweden (CBCS), Science for Life Laboratory, Karolinska Institute, Sweden.
[3]Department of Chemistry-BMC, Science for Life Laboratory, Uppsala University, Sweden.
[4]Center for Molecular Medicine, Department of Medicine (Solna), Science for Life Laboratory, Karolinska Institute, Sweden.
[5]Department of Medical Sciences, Uppsala University, Sweden.
[6]Department of Pharmacy, Uppsala University Drug Optimization and Pharmaceutical Profiling (UDOPP), Science for Life Laboratory, Uppsala University, Sweden.
[7]Institute of Pathology, Neuropathology and Molecular Pathology, Medical University of Innsbruck, Austria.
[8]Diagnostic and Research Institute of Pathology, Medical University of Graz, Austria.

[*]To whom correspondence should be addressed to Rasel A. Al-Amin. Tel: +46700535324; Email**:** drrasel.alamin@gmail.com and to Ulf Landegren. Tel: +46184714910; Fax: +46 18 4714808; Email**:** ulf.landegren@igp.uu.se

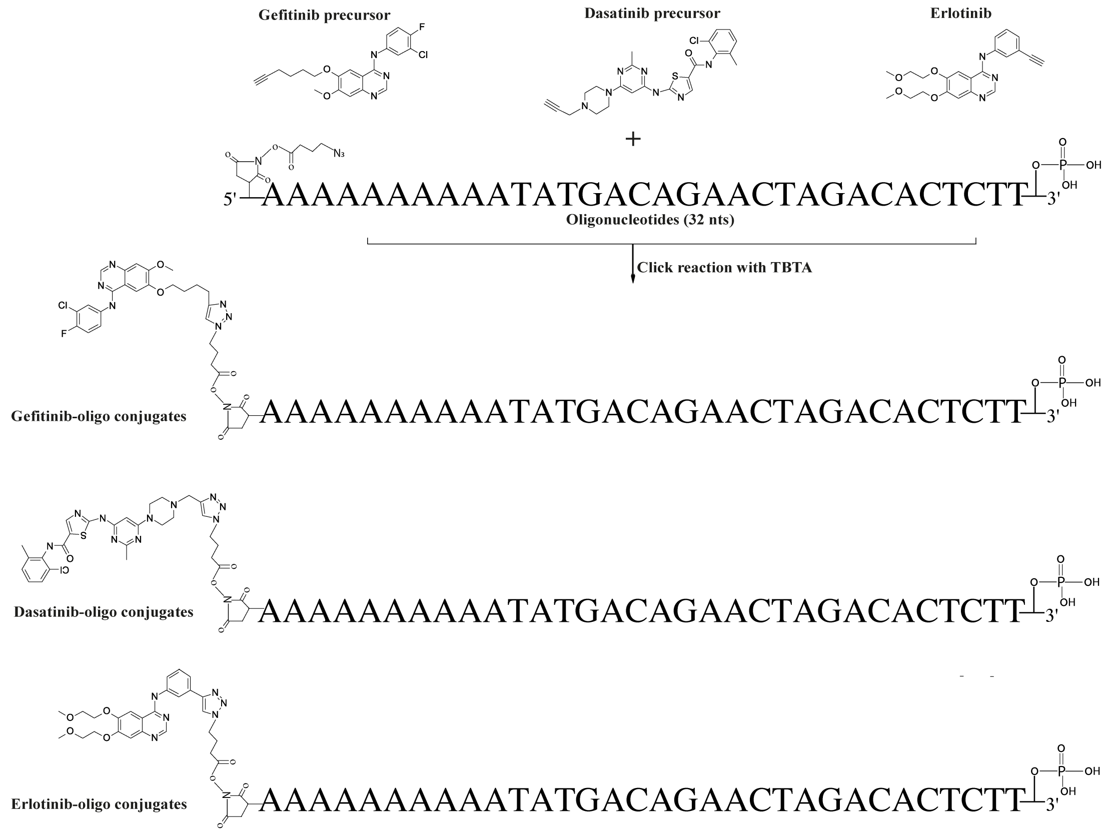

Supplementary Figure 1. Drug-oligonucleotide conjugation reaction. Click chemistry was used to conjugate an alkyne residue in a drug molecule to an azide-modified oligonucleotide. Precursor compounds of gefitinib and dasatinib with added alkyne groups at sites not engaged in target interactions, and native erlotinib having an alkyne function were all conjugated to 5'-azide-modified DNA oligonucleotides using $CuSO_4$ (II) and TBTA-catalyzed click chemistry to form triazole bonds between the DNA oligonucleotides and the low molecular weight drugs.

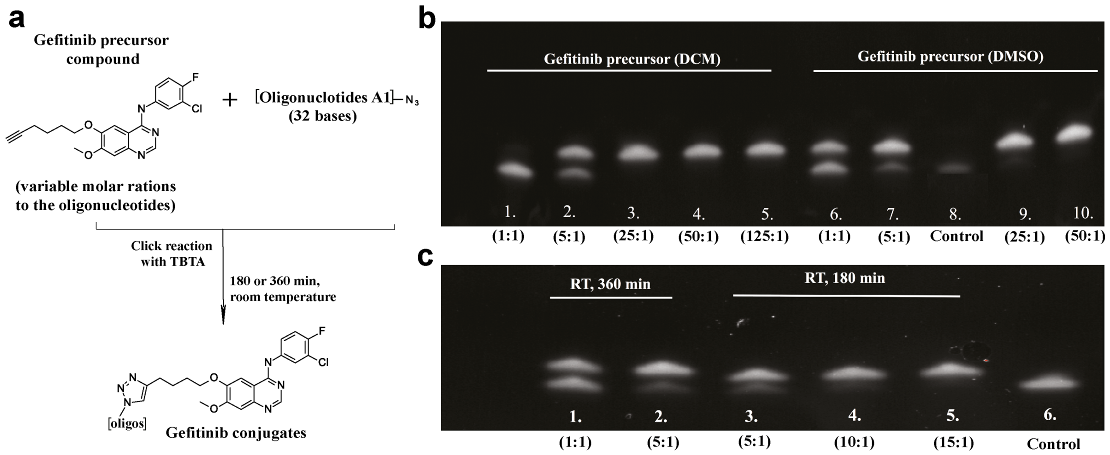

Supplementary Figure 2. Stoichiometry of conjugation reactions and gel validation. a, Click reactions between azide-modified oligonucleotides and gefitinib precursor compounds modified to include an alkyne function, were conducted at room temperature (RT) for 180 min. The click reactions were performed in the presence of $CuSO_4$ (II) and the TBTA catalyst to form a triazole bond. The reactants were added in different molar proportions (1, 5, 10, 15, 25, 50 and 125-fold excess of compound over oligonucleotides). We also investigated and compared the two solvents dichloromethane (DCM) and dimethylsulfoxide (DMSO) for their effects on conjugation efficiency. **b,** Gel validation**:** Lanes 1 and 6 show products from equimolar reactions, indicating that gefitinib precursor compound was more readily conjugated when dissolved in DMSO. The expected shift to a slower-moving species, representing oligonucleotides conjugated with gefitinib precursor compounds, was observed for reactions with the indicated molar ratios between oligonucleotides and gefitinib precursors. Lanes 2, 3, 4, 5, 7, 9 and 10 demonstrate that with a 5-fold or higher molar excess of gefitinib precursor compounds most oligonucleotide shifts to a slower-moving species. The migration of the unconjugated oligonucleotides is seen in lanes 2, 6 and 7. The free oligonucleotide (35 nts in length) was loaded in lane 8 as control. **c,** Reaction kinetics: Conjugation reactions were incubated for 180 or 360 min at the indicated ratios. The results indicate that conjugation at a ratio of 5:1 is slightly more effective using the longer incubation time shows between in lanes 1 and 3. Further the reactions were also tested in 10 and 15-fold molar excess of gefitinib precursor compounds over oligonucleotides in lanes 4 and 5. The reactions were evaluated by gel electrophoresis (Novex® TBE-Urea 15% gel with 1xNovex® TBE urea sample buffer, Invitrogen) running at 100 V constant for 60 min at RT. The gel was stained with SYBR® gold for 20 min in the dark at RT to visualize single-stranded DNA. Images were developed in a BIO-RAD (Molecular image) Gel Dox® XR image station with image lab software.

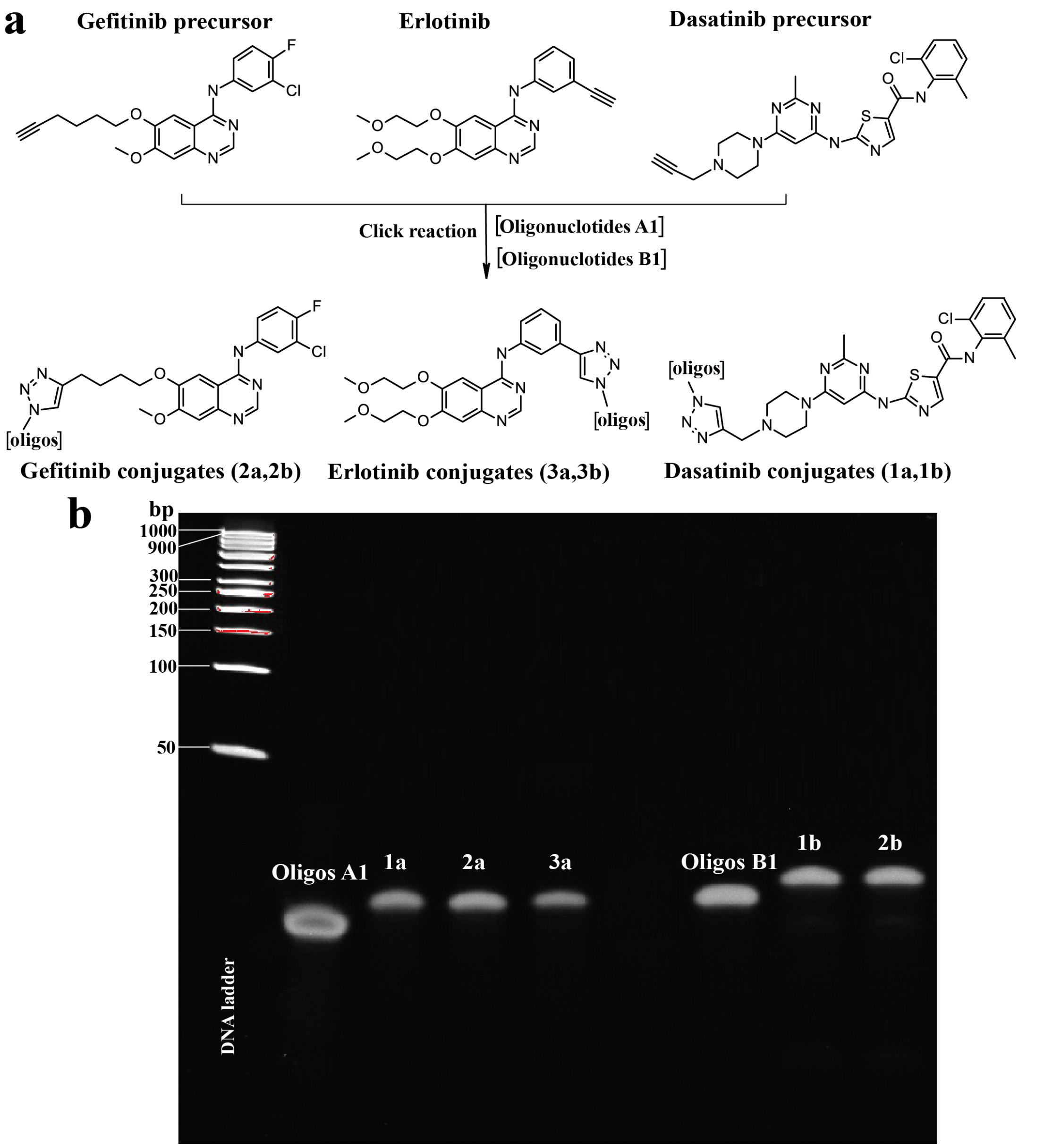

Supplementary Figure 3. Drug-oligonucleotide conjugates and binding characteristics a, Click chemistry was used to conjugate the drug precursors to oligonucleotides. Azide-modified oligonucleotides were combined with a 25-fold molar excess of the precursors of dasatinib or gefitinib, or with native erlotinib, each containing one alkyne residue. The morpholine in gefitinib and dasatinib were replaced with an alkyne and click chemistry afforded conjugation to azide-modified oligonucleotides **A1** (32 nts) and **B1** (35 nts). The corresponding alkyne precursors of dasatinib and gefitinib were synthesized and used to generate dasatnib- and gefitinib-oligonucleotide conjugates **1a** (32 nts), **b** (35 nts) and **2a** (32 nts), **2b** (35 nts), while the alkyne in erlotinib allowed direct conjugation to provide erlotinib-oligonucleotide conjugates **3a** (32 nts) and **3b** (35 nts)**. b,** The conjugation reactions were evaluated by gel separation of conjugates and the free oligonucleotides. Lanes OligoA1 and OligoB1 show the free oligonucleotides (32 and 35 nucleotides), and lanes 1 to 3 shows OligoA1 conjugated to dasatinib (1a), gefitinib (2a) and erlotinib (3a). Lanes 4 and 5 show conjugates of OligoB1 (35 nucleotides) with dasatinib (1b) and gefitinib (2b).

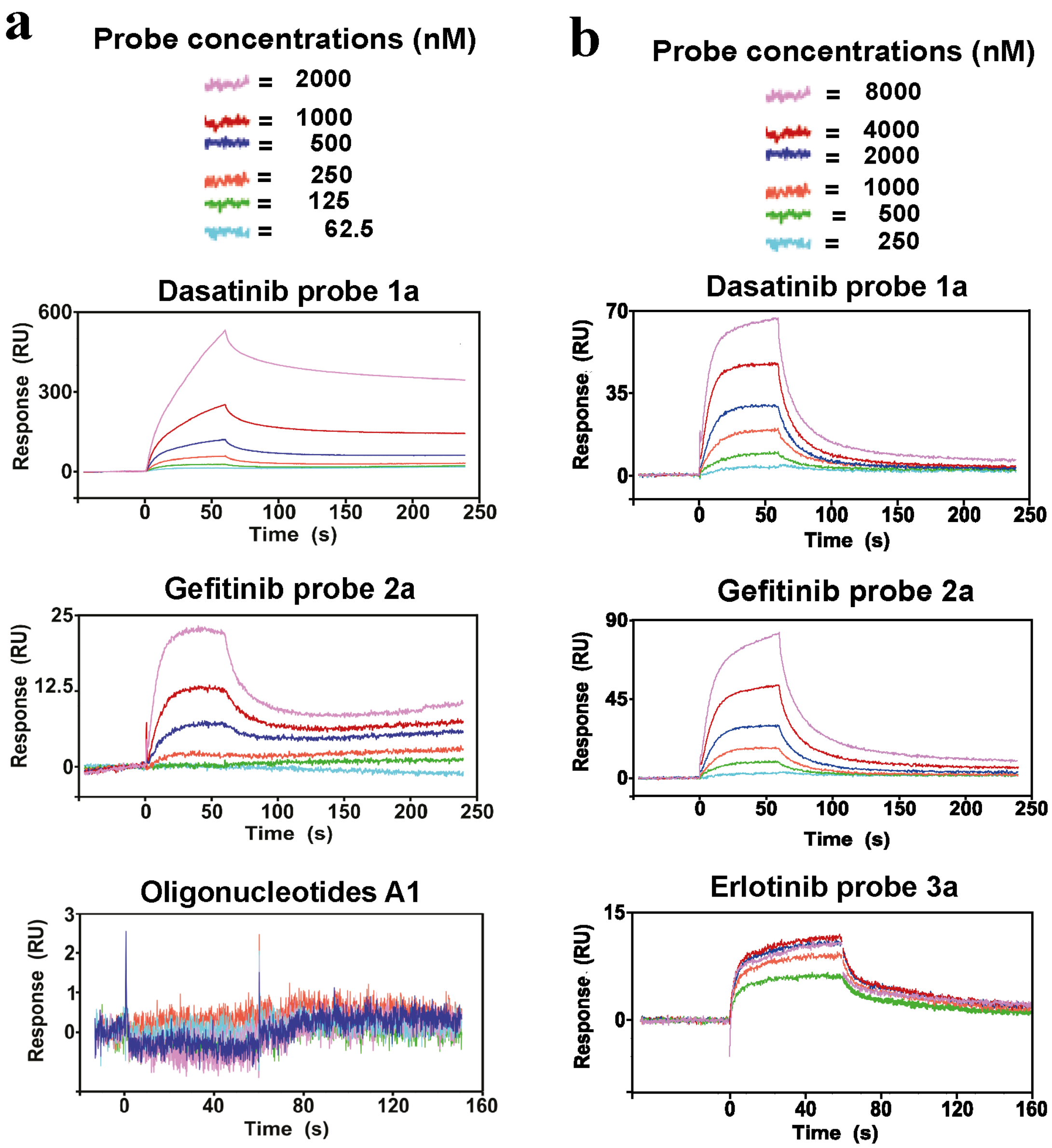

Supplementary Figure 4. Surface plasmon resonance (SPR) biosensor validation of on- and off-target interactions of TEMA probes. a, Sensorgrams of interactions between the oligonucleotide-conjugated dasatinib and gefitinib probes and of azide-modified free DNA oligonucleotides at concentrations from 2000 nM to 62.5 nM with the ABL1 full length protein. A strong on-target interaction with slow off-rate kinetics was observed for the dasatinib probe with one of its primary targets ABL1. The gefitinib probe revealed a weak interaction for its known off-target ABL1, while free oligonucleotides showed no interaction with the protein target. **b,** Analysis of on-target interactions of the gefitinib probe and off-target interactions of the dasatinib probe with the EGFR L858R kinase domain at concentrations from 8 µM to 0.25 µM. The sensorgrams display moderate affinities of the dasatinib and gefitinib probes for the protein target, but little affinity by the inactive erlotinib probe used as experimental control. The sequences of the oligonucleotides are shown in Supplementary Table 1.

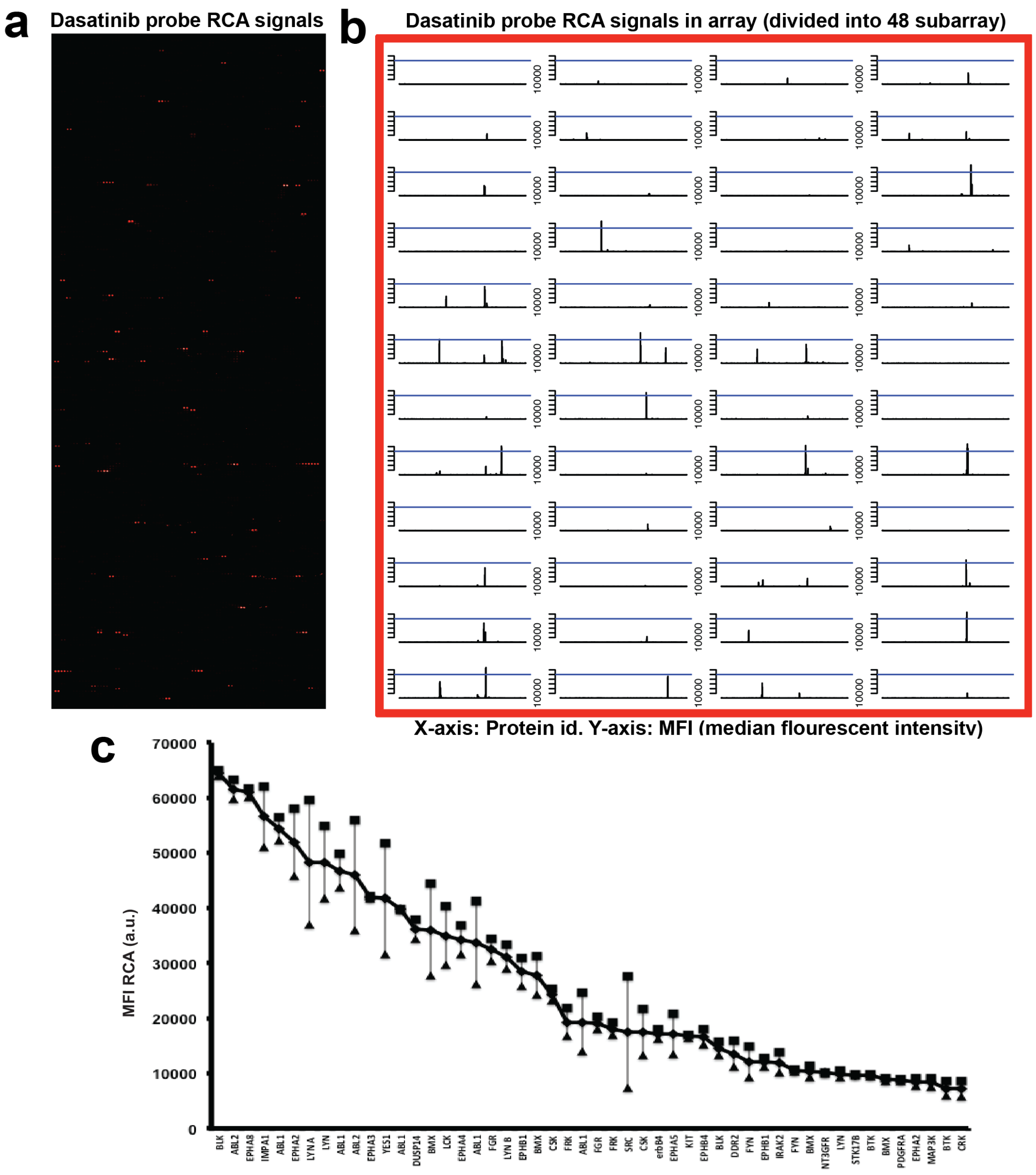

Supplementary Figure 5. TEMA drug profiling in a panel of 9,000 full-length human proteins. a, RCA signals for the dasatinib probe (0.2 nM) added to a protein array, as identified by hits with high median fluorescence intensity (MFI). The oligonucleotide used for detection was conjugated to a FarRed fluorescent reagent (Duolink detection reagents, Sigma Aldrich). **b,** Bar plots show signal profiles within the 48 subarrays, X-axis: protein id and Y-axis: MFI. The maximal Y value was set to MFI 65,535 (laser saturation) and the blue lines indicates a MFI of 50,000. **c,** Signals for the dasatinib probe at 0.2 nM binding to duplicate proteins as identified by TEMA, duplicate measurements and their means are plotted. The results illustrate signals from the 50 proteins yielding the highest values, almost all of them kinases as identified in the hits list.

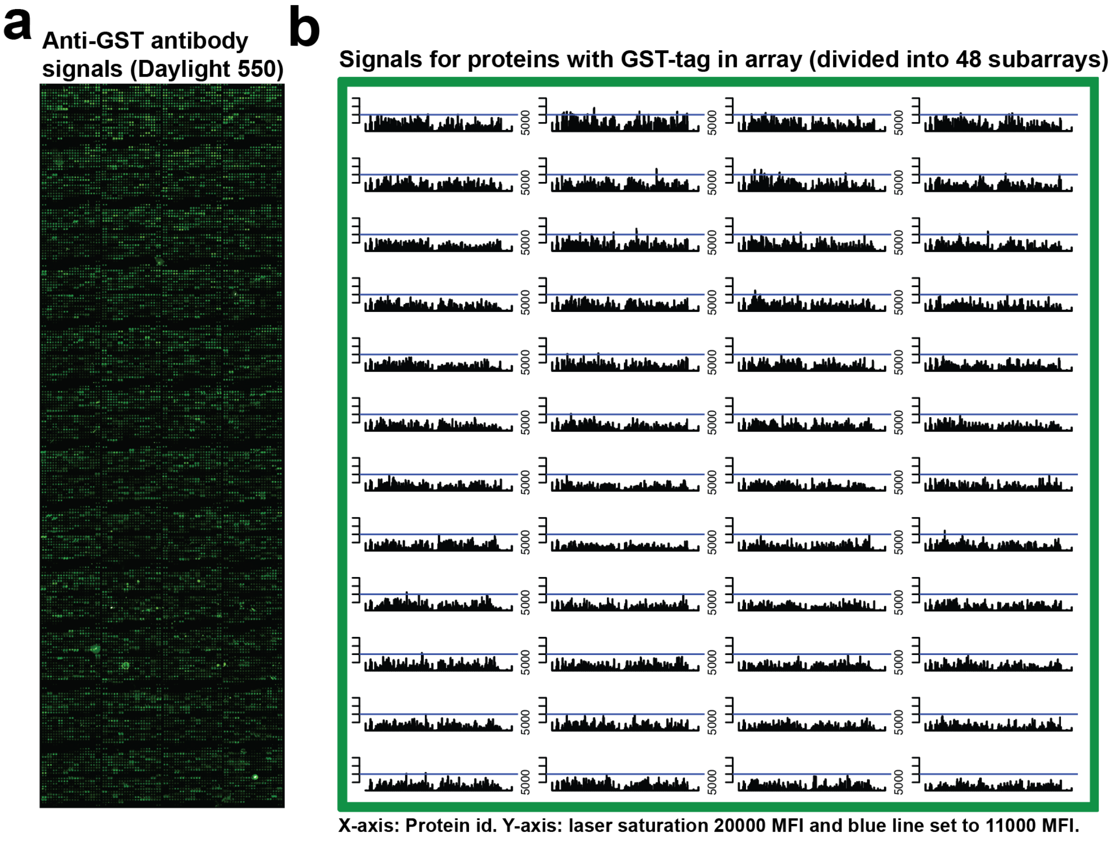

Supplementary Figure 6. Total protein levels in the microarray evaluated by by anti-protein tag staining. a, Detection of protein via their GST-tags, reflecting total amounts of protein per spot in the same array used to evaluate dasatinib binding in figure 2a and supplementary figure 3. The anti-GST antibodies were labeled with DayLight® 550 fluorophores. The green channel was set at 100% laser intensity. **b,** Bar graphs showing signal profiles across the 48 subarrays, reflecting the quality and quantity of proteins spotted in the array. The maximal Y value was set to MFI 65,535 (laser saturation) and the blue lines indicates a MFI of 50,000.

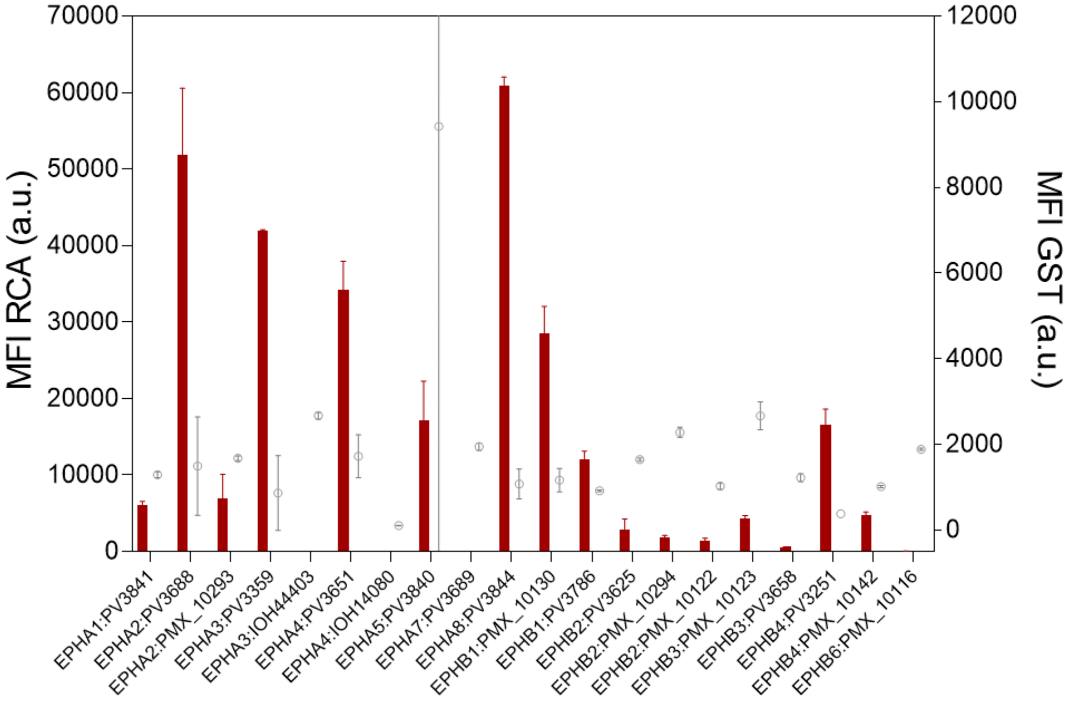

Supplementary Figure 7. Microarray TEMA results for the Ephrin receptor subfamily of tyrosine kinases. Median fluorescence intensity (MFI) signals for the displayed proteins (red; left y-axis) and the corresponding signals for GST (○; right y-axis) – error bars represent difference between two technical replicates. The x-axis labels denote receptor identity combined with the product number of the spotted protein. Large variation was observed when comparing different variants of spotted proteins, as observed for *e.g.* EPHA2 and EPHA4.

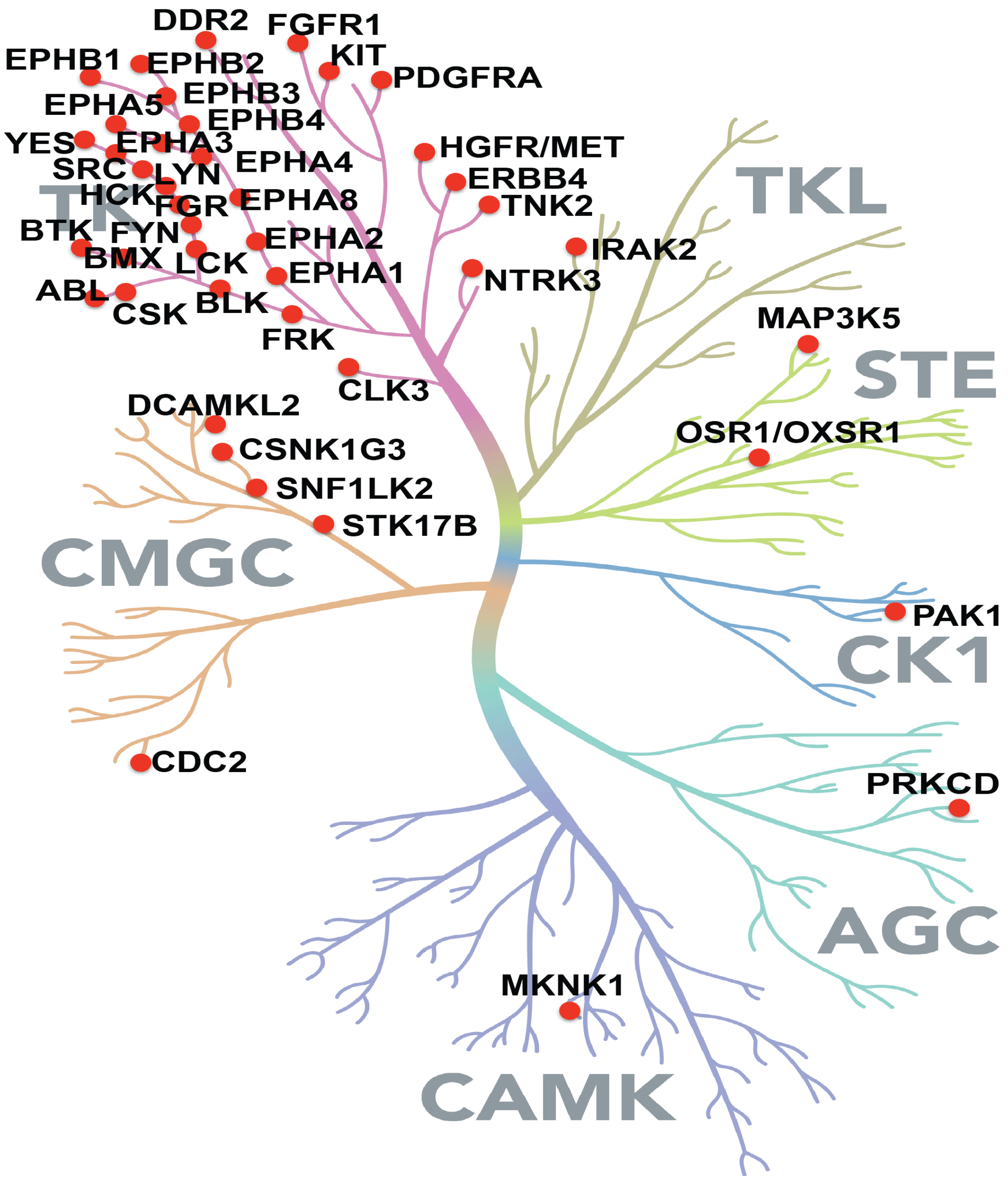

Supplementary Figure 8. Binding in the kinome dendrogram by dasatinib probe as revealed by TEMA. The intensity of TEMA signals by the dasatinib probe plotted in dendrograms that illustrate the relatedness of human kinases. The distribution of kinase TEMA hits (with the cut-off signals above a 2,000 MFI threshold, MFI>2,000) on the human kinome tree for the dasatinib probes among 9,000 total spotted proteins in the array. The human kinome map dendrogram was adapted with permission from Cell Signaling Technology (www.cellsignal.com). Non-receptor tyrosine kinases (NRTK), tyrosine kinase-like kinases (TKL), casein kinase family (CK), protein kinase A, G, and C families (AGC) and calcium/calmodulin dependent kinases (CAMK).

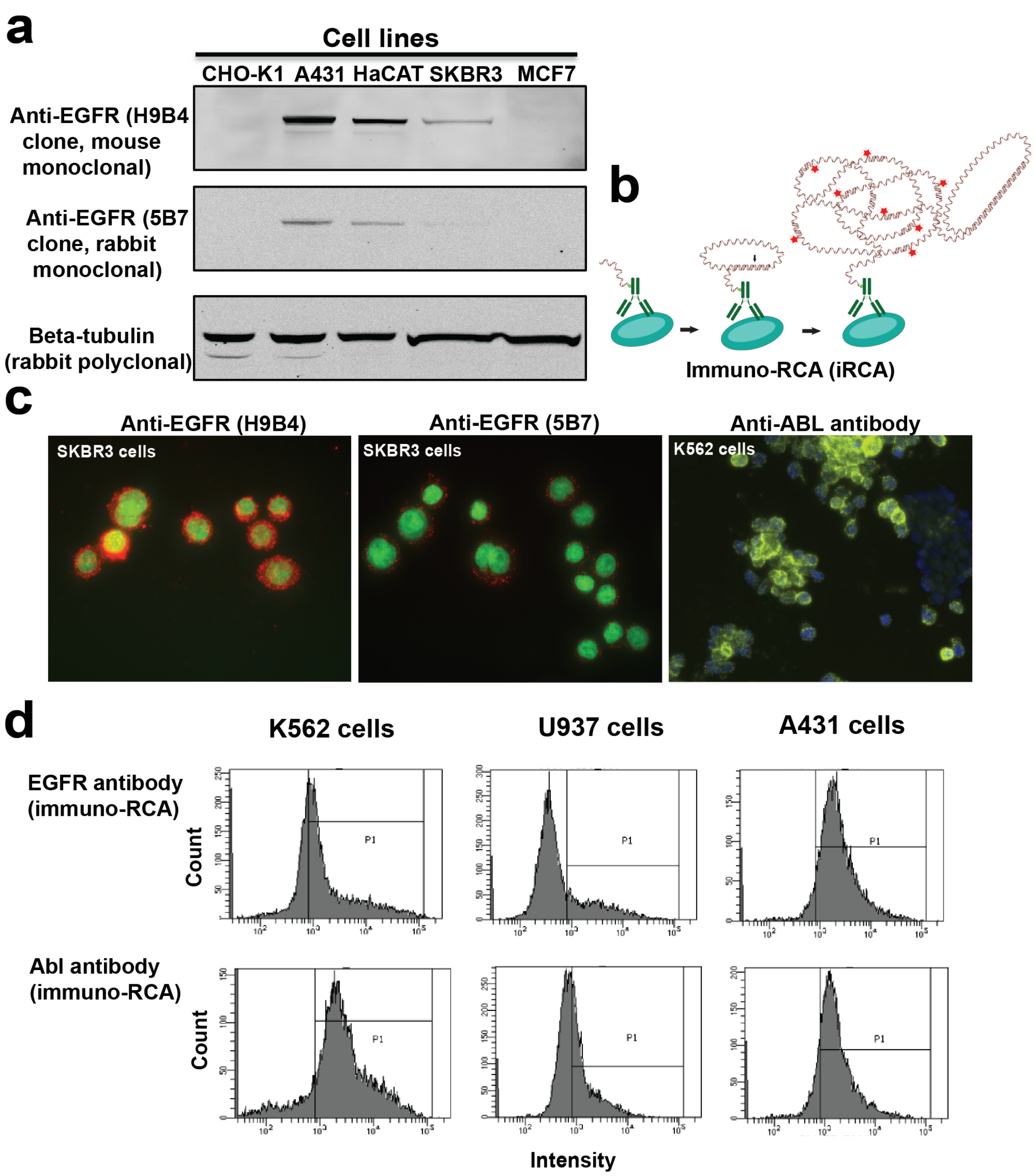

Supplementary Figure 9. Validation of cell lines, antibodies and antibody probes. a, Immunoblots showing EGFR protein expression in five different cell lines CHO-K1, A431, HaCAT, SKBR3 and MCF7. The mouse monoclonal antibody H9B4 is specific for the intracellular (cytoplasmic) domain of the EGFR protein, while the rabbit monoclonal antibody 5B7 is specific for the extracellular portion of EGFR. An anti beta-tubulin antibody was used as a loading control, revealing beta-tubulin protein expression in all cells. **b,** Schematic illustration of iRCA; a single oligonucleotide-modified antibody was used for amplified protein detection, where oligonucleotides on antibodies having bound their targets are used, first to template ligation of an added oligonucleotide to convert this to a single-stranded oligonucleotide circle, and then to prime RCA by replicating the circularized oligonucleotide. **c,** Images of iRCA analyses for validation of binding by anti-EGFR Ab-15 (H9B4), anti-EGFR (5B7) antibody probes in SKBR3 cells and anti-ABL1 antibody probe in K562 cells. The cells were counterstained using DAPI (green or blue) and images were acquired by fluorescence microscopy. **d,** Oligonucleotide-conjugated anti-EGFR (H9B4) or anti-ABL1 antibody probes at 200 ng/ml were used for immune-RCA reaction as positive control detections for flow cytometric readout.

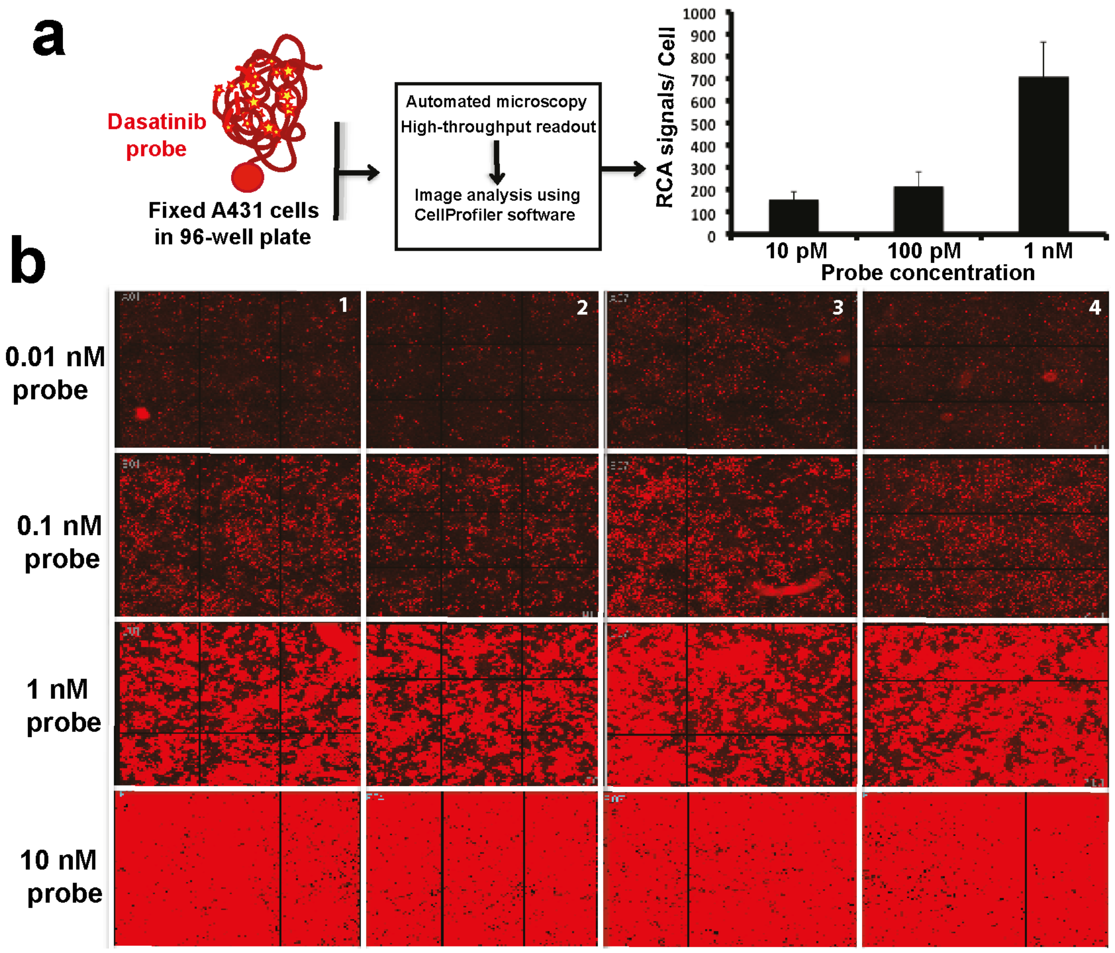

Supplementary Figure 10. Sensitive detection of drug binding in fixed cells. a, The TEMA method was optimized by analyzing A431 cells, cultured and fixed in a 96-well format, recording the results of incubating with a dasatinib probe by high-throughput automated microscopy using an ImageXpress Microplate scanner (Molecular Devices). The signal intensity was quantified per cell using CellProfiler software. The bars show mean values of quadruplicate observation with standard deviation indicated (±). **b,** The concentration of the dasatinib probe was varied as indicated for detecting drug binding in A431 cells by TEMA with staining shown in red. Images in the figure represent low-resolution overviews of 9 images per condition, displayed in a 3 x 3 tile format using the MetaXpress software from four separate experiments (1-4).

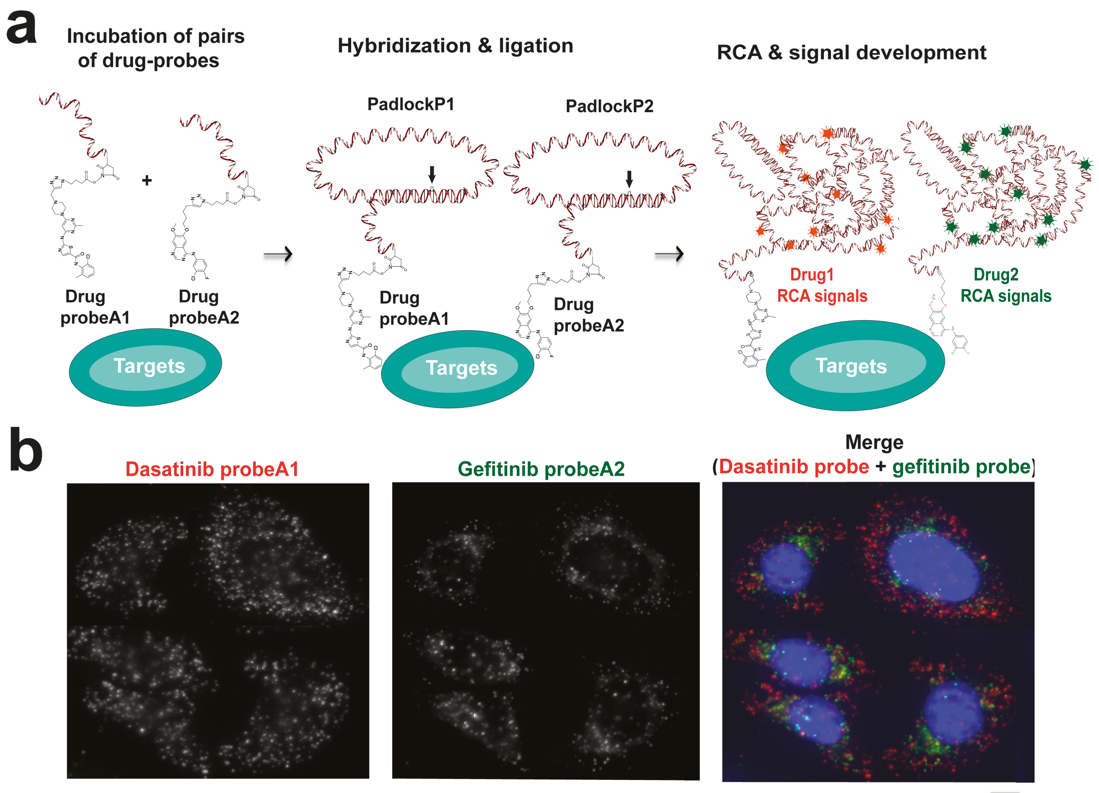

Supplementary Figure 11. Parallel in situ localization of binding by two drugs in cells. a, Oligonucleotide-conjugated dasatinib and gefitinib probes were incubated with fixed and permeabilized SKB3 cells, followed by a brief wash. The two drug-conjugated oligonucleotides then templated ligation by two added padlock probes, which after circularization templated RCA. The local amplification products are detected by hybridization of two oligonucleotide probes labeled with distinct fluorophores. **b,** Parallel visualization of the subcellular localization of dasatinib probes, added at 0.5 nM (red signals), and gefitinib probes, added at 5 nM (green signals). Nuclei have been stained with propidium iodide in the third panel (blue).

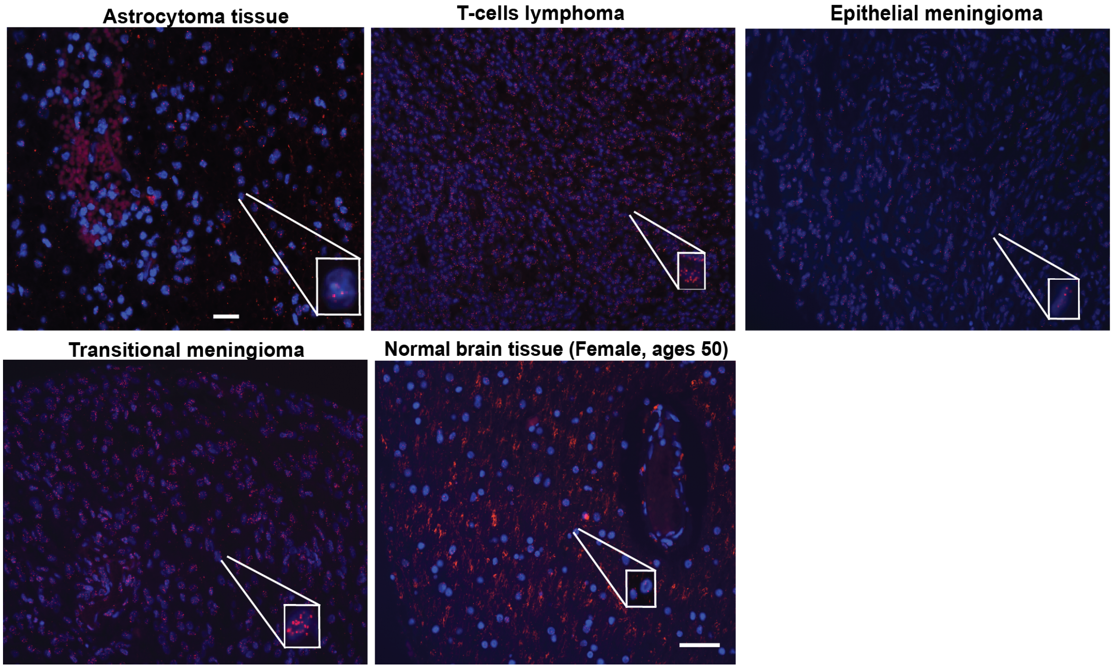

Supplementary Figure 12. Gefitinib probe target engagement in formalin-fixed brain tumor tissue. Investigation of binding by oligonucleotide-conjugated gefitinib probes at 10 nM in arrayed, formalin-fixed paraffin-embedded malignant and normal brain tissue samples. The commercial tissue microarray T175a was from US Biomax. 20x magnified images were acquired using a fluorescence microscope (Zeiss Axioplan2 image station and Zeiss AxioCam camera MRm). Scale bars = 50 µm.

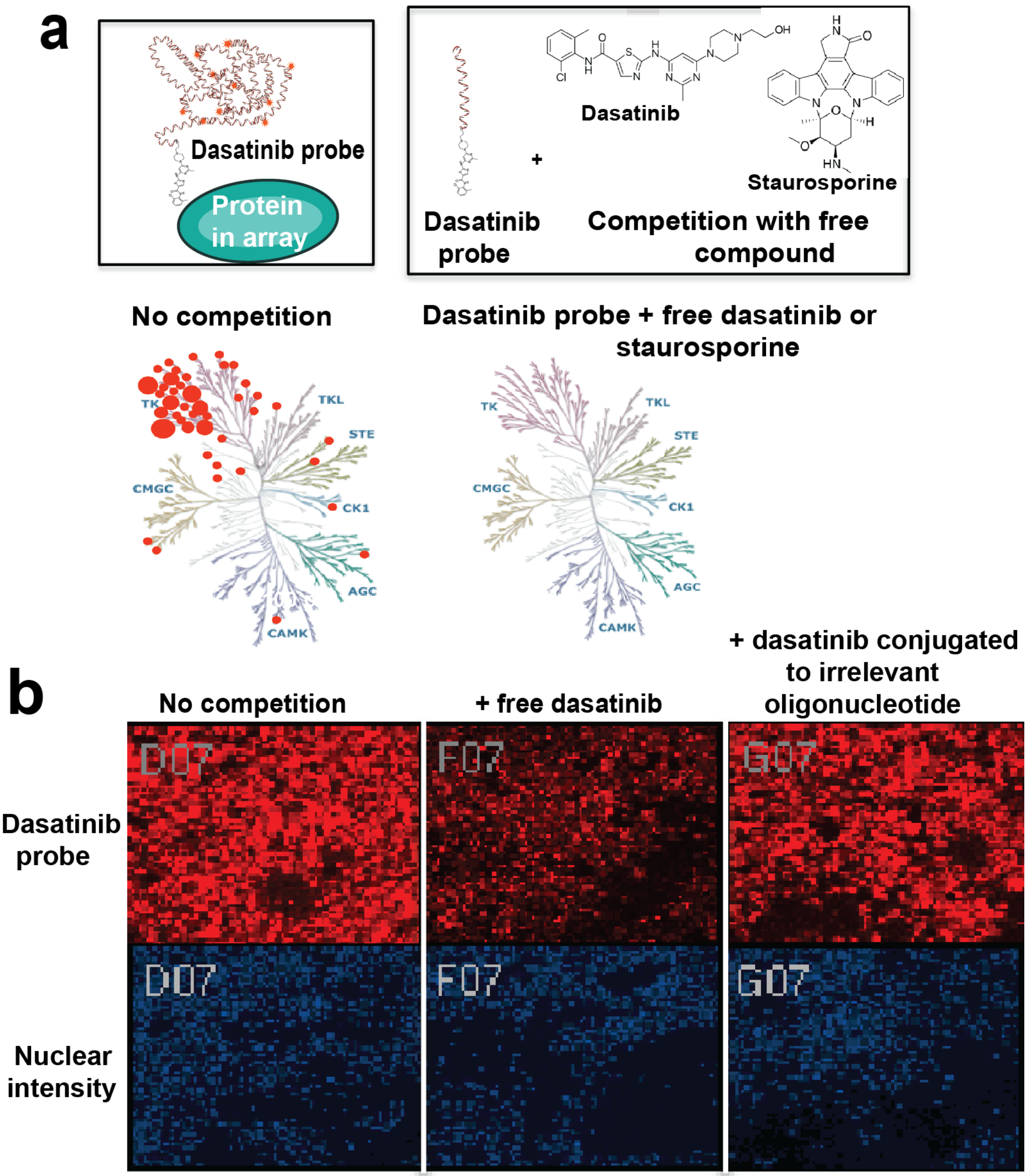

Supplementary Figure 13. Competitive TEMA analysis using oligonucleotide-conjugated or unmodified drug molecules. a, TEMA in competitive binding mode for profiling binding by the dasatinib probe alone or in competition with various kinase inhibitors. An equimolar amount of unmodified dasatinib or staurosporine completely inhibited protein binding (right kinome tree). **b,** In situ TEMA in A431 cells using the dasatinib probe at 1 nM alone or together with the same concentration of unmodified dasatinib or dasatinib conjugated to an irrelevant oligonucleotide (OligoB2). A431 cells were grown in 96-well plates and for each condition nine 20x magnification images were acquired by scanning microscopy. Images in the figure represent low-resolution overviews of a image for each condition using the MetaXpress software. The images were developed by automated microscopy using an ImageXpress Microplate scanner (Molecular Devices).

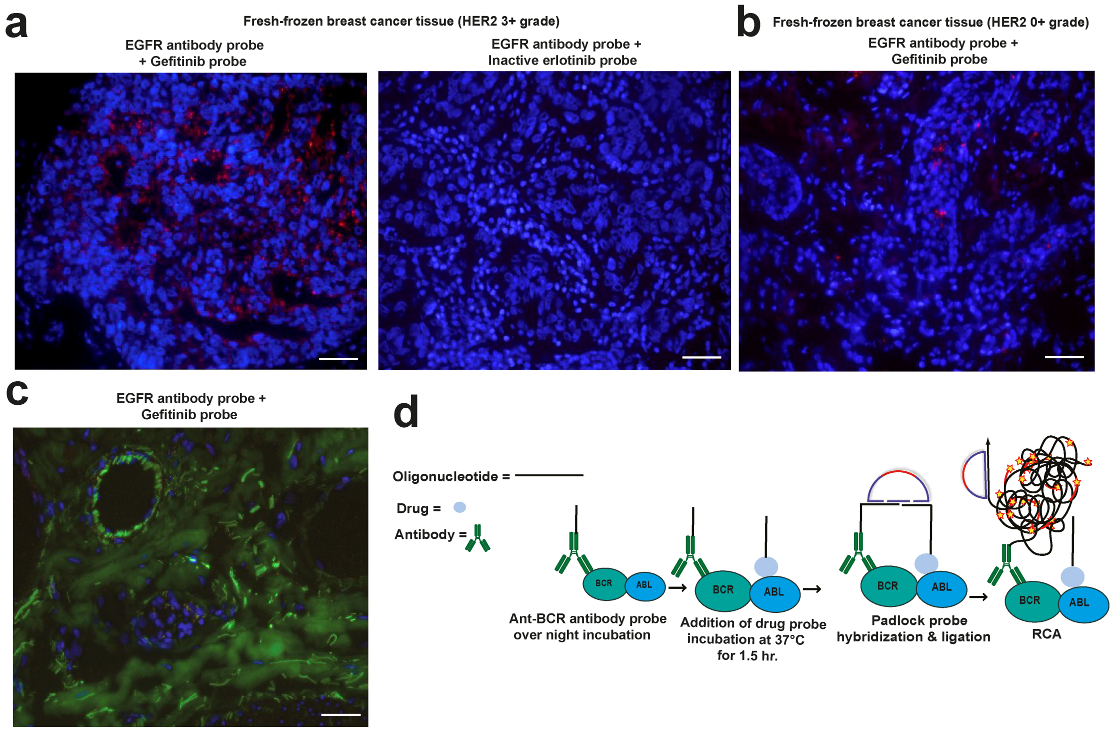

Supplementary Figure 14. In situ analysis of specific interactions of oligonucleotide-conjugated drugs with endogenous proteins via proxTEMA. a, ProxTEMA results using anti EGFR antibody probes together with 10 nM gefitinib or inactive erlotinib probes in a breast cancer tissue section scored as a 3+ grade according to HER2 protein staining. **b,** ProxTEMA results for assays using anti EGFR antibody probes together with 10 nM gefitinib probe in breast cancer tissue sections, scored as 0+ grade according to HER2 protein staining. **c,** Demonstration of negative proxTEMA results for a gefitinib probe at 10 nM and an EGFR-specific antibody probe in normal human fresh-frozen colon tissue sections, expressing low EGFR levels. The tissues were counterstained using DAPI (blue) and images were acquired by fluorescence microscopy. **d,** A schematic of the proxTEMA assay shown in Figure 5b for localized detection of BCR-ABL fusion proteins using oligonucleotide-conjugates of both BCR antibodies and drug molecules. The cells are first incubated with the antibody conjugates and washed before addition of the drug conjugate. After a brief wash to preserve drug binding, RCA is initiated, primed from the antibody-oligonucleotide conjugate. Successful detection depends on proximal binding of the fusion proteins by oligonucleotide conjugates of both drugs and antibodies. Scale bars in panels a-c represent 50 μm

Supplementary Table 1. Oligonucleotide sequences.

Oligonucleotides	Sequences and modifications	Numbers of nucleotides
**OligoA1**	5'-azide-AAAAAAAAAATATGACAGAACTAGACACTCTT-3'	32
**OligoA2**	5'-azide-AAAAAAAAAATATGACAGAACATACGGTCTCG CAGATCGCTTAGACACTCTT-3'	52
**OligoB1**	5'-Azide-AAAAAAAAAAGACGCTAATAGTTAAGACGCTT UUU-3'	35
**OligoB2**	5'-azide-GACGCTAATAGTTAAGACGCTTUUU-3'	25
**PadlockP1**	5'-phosphate-GTTCTGTCATACAGTGAATGCGAGTCCGTCTA AGAGAGTAGTACAGCAGCCGTCAAGAGTGTCTA-3'	65
**PadlockP2**	5'-phosphate-ATGATCGTCCTTATCACTGCCCCGACAGGCCT CAGACATCATAATAGCTCCTAGTGCTGG-3	60
**PadlockP3**	5'-phosphate-GAGACCGTATCGATCACTGCCCCGACAGGCCT CAGACATCATAATAGCGTAGCGATCTGC-3	60
**Detection oligonucleotideD1**	5'-Texas Red/FarRed/ Cy3/HRP- CAGTGAATGCGAGTCCGTCTmUmUmUmU-3'	20
**Detection oligonucleotideD2**	5'-Texas Red/FITC- AGCGATCTGCGAGACCGTATmUmUmUmU**-3'	20
**Detection oligonucleotideD3**	5'- FarRed/FITC/Cy3/HRP - CTAGTGCTGGATGATCGTCCmUmUmUmU-3'	20

**mU = 2' O-Methyl RNA bases added for protection against degradation by 3' exonuclease activity

Supplementary Table 2. Nominal mass LC-MS measurements.

Compounds	Calculated Mw (Da)	Observed Mw (Da)	Daughter ion 1
Dasatinib precursor	482	482.012	400.09
Gefitinib precursor	399	400.032	319.988
Erlotinib	393	394.096	336

Oligonucleotides	Mwt (Da) (average)	Observed ion 1 (m/z)	Predicted ion 1 (m/z)
OligoA1	10148	844.6	844
OligoA2	16307.8	857	857
OligoB1	11079.4	851	851
OligoB2	7077	1009	1010

Conjugates	Drug-oligo conjugate	Observed ion 1 (m/z)	Predicted ion 1 (m/z)	Observed ion 2 (m/z)	Predicted ion 2 (m/z)
Dasatinib-OligoA1	10629	884	884	816	816
Gefitinib-OligoA1	10547	878	877	810	810
Erlotinib-OligoA1	10541	877	877	810	810
Dasatinib-OligoA2	16790	882	882	838	838
Gefitinib-OligoA2	16707	878	878	834	834
Erlotinib-OligoA2	16701	877	878	833	834
Dasatinib-OligoB1	11561	888	889	824	824
Gefitinib-OligoB1	11479	881	882	819	819
Erlotinib-OligoB1	11473	881	882	818	818
Dasatinib-OligoB2	7559	1078	1078	943	943
Gefitinib-OligoB2	7476	1067	1067	933	933
Erlotinib-OligoB2	7470	1066	1066	932	933

TEMA	Competition binding assay		Kinobeads assay		Activity-based kinase assays		
**Ranking of gene in array (0.2 nM)**	**Davis et al., 2011. Kd [nM]**	**Karaman et al., 2008. K_D [nM]**	**Medard et al., 2015. Kd/IC50 [nM]**	**Bantscheff et al., 2007. IC_{50} [nM]**	**Kitagawa et al., 2013. Inhibition [%]/IC_{50} [nM]**	**Anastassia et al., 2011. Residual activity [%]**	**Rix et al., 2008. Seq. coverage (%)**
1. BLK	0.21	0.21			103.00 / 0.44	3.0	
2. ABL2	0.17	0.17	1.2/10	31	102.1/0.44	2.8	19
3. EPHA8	0.24	0.24			100.00/0.32	4.1	
5. ABL1	0.047	0.53	2.6/6.8	45	104.80/0.27	2.8	29
6. EPHA2	0.85	0.85	2.8/13		99.40/1.6	6.8	
7. LYN A					100.5/0.28		
8. LYN	0.57	0.57	6.7/21	182	/0.28	0.4	47
9. EPHA3	0.093	0.093			100.10/1.3	3.5	
10. YES1	0.3	0.3	1.8/10	105	101.80/0.18	1.9	23
12. BMX	1.40	1.4			100.50/0.64	2.5	20
13. LCK	0.2	0.2	4.3/16		101.800/0.17	0.3	6
14. EPHA4	1.2	1.2	1.0/8.3	>10,000	98.90/1.7	1.6	
15. FGR	0.5	0.5	6.2/12		103/0.67	1.7	50
16. LYN B					101.60/0.28	1.6	
17. EPHB1	0.45	0.45		>10,000	100.10/1.4	4.8	
18. CSK	1	1	21741	145	103.80/3.2	7.1	59
19. FRK	0.31	0.31	5.0/21	>10,000	100.10/0.19	-0.9	7
20. SRC	0.21	0.21	0.9/4.7	62	101.40/0.19	3.5	40
21. ERBB4	55.00				103.40/3.9	4.0	
22. EPHA5	0.24	0.24	0.7/6.4		100.90/0.44	0.2	
23. KIT	0.81	0.62	40/63	551	99.80/3.3	3.2	
24. EPHB4	0.34	0.34	2.7/7.5	14	100.20/0.51	1.2	13
25. DDR2	3.2	3.2	118/118	>10,000	101/0.9	0.2	
26. FYN	0.79	0.79	0.8/7.6	199	100.10/0.2	2.0	16
28. NTRK3					/1500		
29. BTK	1.4	1.4	4.4/15	54	101.60/0.3	2.1	72
30. PDGFRA	0.47	0.47			99.50/2.6	1.8	
31. EPHA1	4.1	4.1			101/0.65	2.7	
32. EPHB3	6.9	6.9	22/39	>10,000	99.50/2.5	1.0	
33. TNK2	5.6	5.6	5.8/53	475	97/3.3	2.6	
34. HCK	0.35	0.35	26/36	439	99.60/0.16	0.8	56
35.SNF1LK2/	6.4	6.4	5.8/50	152	100.6/5.4	1.9	10
36. EPHB2	0.39	0.39	2.3/7.8	339	99.90/1	-0.2	5

Supplementary Table 3. Comparison of kinase hits for the dasatinib probe by TEMA in protein arrays, along with published results from others approaches(1-8). The left hand column identifies protein array hits by TEMA using the dasatinib probe. The results are consistent with the corresponding data reported in seven other studies, using a variety of assays for assessing target engagement by kinase inhibitors.

Supplementary Table 4. % of signals generated through binding of the dasatinib probe (0.2 nM) in competition with equimolar amounts of unmodified kinase inhibitors (1-8).

Dasatinib probe hits	Dasatinib probe + unmodified dasatinib	Dasatinib probe + unmodified staurosporine	Dasatinib probe + unmodified bosutinib	Dasatinib probe + unmodified ibrutinib
1. ABL2-onco	0.09	0.1	0.1	34.8
2. ABL1	0.1	0.1	0.2	8.6
3. EPHA3	0.1	0.1	2.9	0.1
4. IMPA1	0.08	0.08	45.2	21.2
5. BMX	0.3	0.5	52.6	7.6
6. BMX (c)	0.1	0.4	26.5	0.7
7. EPHA8	0.07	0.07	0.1	2.3
8. ABL2	0.4	0.5	10.3	33.9
9. EPHA2	0.3	0.4	4.7	0.5
10. YES1 (v)	0.1	0.1	72.9	39.0
11. KIT (v)	0.1	0.1	97.0	70.0
12. EPHB4	0.5	0.5	6.5	0.5
13. BLK	0.2	0.18	112.0	15.0
14. BTK	0.5	0.4	0.6	0.5
15. EPHB1	0.2	0.2	35.6	17.9
16. FYN	0.3	0.4	28.03	16.5
17. FGR	0.2	0.2	9.6	9.1
18. FGR (v)	0.1	0.1	23.0	21.2
19. LYN A	0.09	0.09	0.09	0.09
20. LYN B	0.1	0.1	28.7	19.1
21. LCK	0.1	0.3	10.9	28.1
22. EPHA4	0.1	0.1	10.9	0.8
23. FRK	0.2	0.2	0.7	1.1
24. FRK (Ty)	0.2	0.2	0.3	1.5
25. LYN (v)	0.2	0.2	0.3	0.2
26. TRAP	0.7	0.7	0.8	0.7
27. MET	1.5	1.5	1.6	1.4
28. SRC	0.07	0.07	62.6	45.2
29. CSK	0.2	0.2	4.0	6.1
30. DDR2	0.1	0.1	78.6	32.1
31. erbB-4	0.2	0.2	1.8	1.9
32. ERBB4 (v)	2.7	2.8	3.0	2.5
33. NTRK3	0.4	0.4	3.5	0.4
34. PDGFRA	1.8	1.9	18.6	1.8
35. PDGFRB	0.6	0.6	29.1	0.7
36. CRK (v)	0.55	0.6	2.6	1.3
37. EPHA5	0.23	0.2	19.6	5.1
38. MAP3K5	0.45	0.5	0.5	0.5
39. EPHA1	0.61	0.7	0.8	0.6
40. HCK	1.15	1.2	1.3	1.2

REFERENCES

1. Fabian, M. A.; Biggs, W. H.; 3rd, Treiber, D. K.; Atteridge, C. E.; Azimioara, M. D.; Benedetti, M. G.; Carter, T. A.; Ciceri, P.; Edeen, P. T.; Floyd, M.; Ford, J. M.; Galvin, M.; Gerlach, J. L.; Grotzfeld, R. M.; Herrgard, S.; Insko, D. E.; Insko, M. A.; Lai, A. G.; Lélias, J. M.; Mehta, S. A.; … Lockhart, D. J. (2005) A small molecule-kinase interaction map for clinical kinase inhibitors. *Nat. Biotechnol.*, **23**, 329-336.

2. Ghoreschi, K.; Laurence, A.; O'Shea, J. J. (2009) Selectivity and therapeutic inhibition of kinases: to be or not to be?. *Nat. Immuno.*, **10**, 356-360.

3. Davis, M. I.; Hunt, J. P.; Herrgard, S.; Ciceri, P.; Wodicka, L. M.; Pallares, G.; Hocker, M.; Treiber, D. K.; Zarrinkar, P. P. (2011) Comprehensive analysis of kinase inhibitor selectivity. *Nat. Biotechnol.*, **29**, 1046-1051.

4. Bantscheff, M.; Eberhard, D.; Abraham, Y.; Bastuck, S.; Boesche, M.; Hobson, S.; Mathieson, T.; Perrin, J.; Raida, M.; Rau, C.; Reader, V.; Sweetman, G.; Bauer, A.; Bouwmeester, T.; Hopf, C.; Kruse, U.; Neubauer, G.; Ramsden, N.; Rick, J.; Kuster, B.; … Drewes, G. (2007) Quantitative chemical proteomics reveals mechanisms of action of clinical ABL kinase inhibitors. *Nat. Biotechnol.*, **25**, 1035–1044.

5. Kitagawa, D.; Yokota, K.; Gouda, M.; Narumi, Y.; Ohmoto, H.; Nishiwaki, E.; Akita, K.; Kirii, Y. (2013) Activity-based kinase profiling of approved tyrosine kinase inhibitors. *Genes to cells*, **18**, 110–122.

6. Anastassiadis, T.; Deacon, S. W.; Devarajan, K.; Ma, H.; Peterson, J. R. (2011) Comprehensive assay of kinase catalytic activity reveals features of kinase inhibitor selectivity. *Nat. Biotechnol.*, **29**, 1039–1045.

7. Remsing Rix, L. L.; Rix, U.; Colinge, J.; Hantschel, O.; Bennett, K. L.; Stranzl, T.; Müller, A.; Baumgartner, C.; Valent, P.; Augustin, M.; Till, J. H.; Superti-Furga, G. (2009) Global target profile of the kinase inhibitor bosutinib in primary chronic myeloid leukemia cells. *Leukemia*, **23**, 477-485.

8. Chidley, C.; Haruki, H.; Pedersen, M. G.; Muller, E.; Johnsson, K. (2011) A yeast-based screen reveals that sulfasalazine inhibits tetrahydrobiopterin biosynthesis. *Nat. Chem. Biol.*, **7**, 375-383.

Supporting Materials and Methods

Title: Monitoring Drug-Target Interactions through Target Engagement-Mediated Amplification on Arrays and *in situ*

Supporting Material S1. Reagents. Dasatinib (Sprycel), Gefitinib (Iressa), Erlotinib (Tarceva) and Staurosporine were purchased from Cell Signaling, Bosutinib was purchased from Sigma Aldrich, Ibrutunib (PCI-32765) was purchased from Selleckchem. All compounds were dissolved in DMSO (from Sigma-Aldrich) at 10 mM. Precursor compounds were synthesized with an alkyne modification in a side chain of the compound to allow for conjugation via click chemistry. Water, TBS and PBS were purified with Nalgene Rapid-Flow Filters (Thermo Scientific). Oligonucleotides were purchased from Integrated DNA Technologies (IDT), oligonucleotide sequence compositions and modifications are reported in Supplementary Table 1. Oligonucleotide concentrations were measured by UV spectroscopy using a Nanodrop ND1000 spectrophotometer and they were analyzed by PAGE (15% TEB-urea gel (Invitrogen). Further characterization was performed by reverse-phase high-pressure liquid chromatography (HPLC) and LC/ESI-MS from Waters XEVO TQ MS coupled to an Acquity UPLC.

Cell Culture. The cell lines A431 (human epidermoid carcinoma cells), CHO-K1 (chinese hamster ovary derived cells), HaCAT (human keratinocytes cells), and MCF7 (human breast adenocarcinoma cells) were cultured in DMEM (Invitrogen, US), while K562 (human erythroleukemic cells), SK-BR-3 (breast cancer cells) and U937 (histiocytic lymphoma cells) were grown in RPMI1640 medium. All media were supplemented with 10% fetal bovine serum (FBS, Gibco), 2 mM L-glutamine and 1% or $100 \mu U \mu ml^{-1}$ penicillin-streptomycin (all reagents from Sigma Aldrich). Cells were grown at 37°C in a humidified air incubator with 5% CO_2, and routinely sub-cultured twice a week using 0.5% trypsin-EDTA. The medium from cells grown on Lab-Tek chamber slides (Nalge Nunc International) medium was aspirated, the cells were washed with 1x PBS and fixated with 3.7% PFA on ice for 20 min. Cells were washed twice in DEPC-treated PBS after fixation and dehydrated in an ethanol series of 70%, 85% and 100% for 5 min each and stored at -20°C. Cell slides were removed from storage at -20°C and washed with PBS, permeabilized with TBS-0.02% Triton for 20 min. After permeabilization, cells were washed twice in DEPC-treated PBS. For flow cytometry experiments, the U937 and K562 cells were removed from the media by centrifugation and washed once in 1x PBS prior to fixation. A431 cells were trypsinized (0.25% trypsin/EDTA from Gibco) and then washed once in 1x PBS. The cells were fixated in 1% Formaldehyde Solution (Sigma Alrich) in 1x PBS in 4°C over night. After incubation the fixation solution was removed and the cell pellets were washed in PBS before permeabilization by resuspending the cells in 0.25% Saponin and 0.1% Triton X-100 (both from Sigma-Aldrich) in 1x PBS and incubated for 30 min at RT.

Supporting Methods S2. Design, synthesis and characterization of clickable dasatinib and gefitinib precursor compounds. From literature structure-activity relationship and X-ray co-crystal structure studies, chemical modifications were introduced at the positions indicated by arrows in dasatinib (Sprycel) and gefitinib (Iressa) at the morpholino substituent (1). The sites of attachment were selected to minimize risk of interference with target protein binding by the conjugated molecules (2,3). For erlotinb (Tarceva) it is known that the placement of a large substituent, such as the oligonucleotide used here, is not tolerated for EGFR inhibition, and thereby renders the conjugate unable to bind its target (4).

Supporting Methods S3. Structure, synthesis and characterization of precursor compounds. All commercial reagents and solvents were used without further purification. 4-[(3-chloro-4-fluorophenyl)amino]-7-methoxyquinazolin-6-ol and 2-[(6-chloro-2-methylpyrimidin-4-yl)amino]-*N*-(2-chloro-6-methylphenyl)-1,3-thiazole-5-carboxamide were purchased from Ark Pharma. Preparative HPLC was performed on a Gilson HPLC system at either basic or acidic pH. Basic pH: Column Xbridge Prep C18, 5 μM CBD (30 × 75 mm); 50

mM NH_4HCO_3, pH 10 and acetonitrile were used as mobile phases at a flow rate of 45 ml/min, with a gradient time of 9 min. Acidic pH: Column ACE 5 C8 (150 mm × 0.3 mm); H_2O (containing 0.1% TFA) and acetonitrile were used as mobile phases at a flow rate of 45 ml/min, with a gradient time of 9 min. ^{1}H NMR spectra were recorded on a Bruker DRX-400 NMR spectrometer. Chemical shifts are expressed in parts per million (ppm) and referenced to the residual solvent peak. Analytical HPLC-MS was performed on an Agilent MSD mass spectrometer connected to an Agilent 1100 HPLC system using methods A and B. Method A (acidic pH): column ACE 3 C8 (50 × 3.0 mm); H_2O (+ 0.1% TFA) and acetonitrile were used as mobile phases at a flow rate of 1 ml/min, with a gradient time of 3.0 min. Method B (basic pH): column X-Terra MSC18 (50 × 3.0 mm); 10 mM NH_4HCO_3 pH 10 and acetonitrile were used as mobile phases at a flow rate of 1 ml/min, with a gradient time of 3.0 min. HPLC-MS detection was performed by UV using 180–305 nM wavelength range and MS (ESI+). All final compounds were assessed to be >95% pure by HPLC-MS UV analysis.

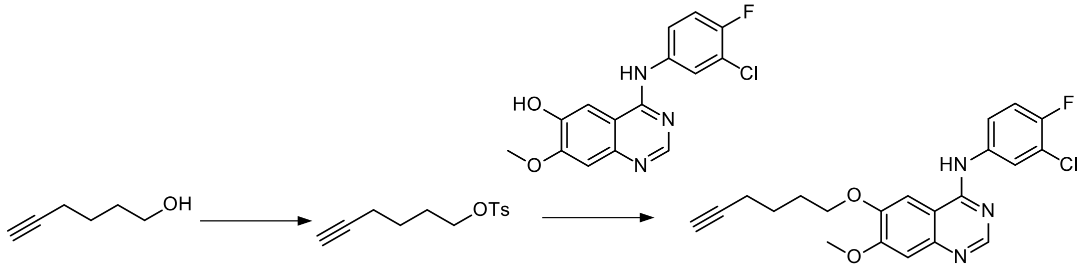

***N*-(3-Chloro-4-fluorophenyl)-6-(hex-5-yn-1-yloxy)-7-methoxyquinazolin-4-amine.** 4-methylbenzene-1-sulfonyl chloride (119 mg, 0.63 mmol) was added to an ice-cool solution of hex-5-yn-1-ol (0.86 ml, 0.78 mmol), triethylamine (0.11 mL, 0.78 mmol) and *N*,*N*-dimethylpyridin-4-amine (7.6 mg, 0.063 mmol) in dichloromethane (2 ml). After stirring the mixture at room temperature overnight, the solvent was evaporated. With the aid of DMSO (2 mL), the remaining material was transferred to a microwave reactor tube loaded with 4-[(3-chloro-4-fluorophenyl)amino]-7-methoxyquinazolin-6-ol (100 mg, 0,31 mmol) and K_2CO_3 (108 mg, 0.78 mmol). The reaction mixture was heated by microwave irradiation at 100°C for 1 h. After cooling to rt, the resulting suspension was filtered, and the filtrate was purified by preparative HPLC at basic pH to yield the product as an off-white solid (125 mg, 50%). ^{1}H NMR (acetonitrile-*d*3) δ 1.69–1.76 (m, 2 H), 1.94–2.00 (m, 2 H), 2.20 (t, ^4J(H–H) = 2.7 Hz, 1 H), 2.31 (dt, ^3J(H–H) = 7.2 Hz, ^4J(H–H) = 2.7 Hz, 2 H), 3.96 (s, 3 H), 4.18 (t, ^3J(H–H) = 6.5 Hz, 2 H), 7.20 (s, 1 H), 7.25 ("t", ^3J(H–F) = 9.1 Hz, ^3J(H–H) = 9.1 Hz, 1 H), 7.34 (s, 1 H), 7.66 (ddd, ^3J(H–H) = 9.1 Hz, ^4J(H–F) = 4.3 Hz, ^4J(H–H) = 2.8 Hz, 1 H), 8.07 (br. s, 1 H), 8.12 (dd, ^4J (H–F) = 6.8 Hz, ^4J(H–H) = 2.8 Hz, 1 H), 8.49 (s, 1 H).

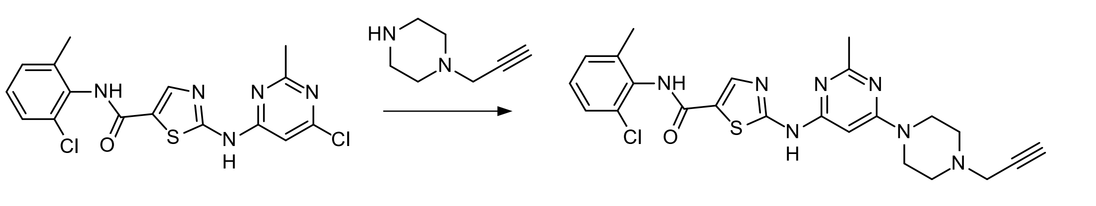

***N*-(2-chloro-6-methylphenyl)-2-({2-methyl-6-[4-(prop-2-yn-1-yl)piperazin-1-yl]pyrimidin-4-yl}amino)-1,3-thiazole-5-carboxamide.** To a suspension of 2-[(6-chloro-2-methylpyrimidin-4-yl)amino]-*N*-(2-chloro-6-methylphenyl)-1,3-thiazole-5-carboxamide (40 mg, 0.10 mmol) and 1-(prop-2-yn-1-yl)piperazine dihydrochloride (50 mg, 0.25 mmol) in 1,4-dioxane (0.2 ml) was added *N*,*N*-diisopropylethylamine (0.18 ml, 1.0 mmol). The mixture was stirred under reflux overnight. The material obtained was purified by preparative HPLC at basic pH to yield the product as an off-white solid (28 mg, 49%). ^{1}H NMR (DMSO-d_6) δ 2.23 (s, 3 H), 2.41 (s, 3 H), 2.50–2.56 (m, obscured by solvent signal, 2 H), 3.19 (m, 1 H), 3.31–3.35 (m, obscured by H_2O signal, 4 H), 3.51–3.58 (m, 4 H), 6.06 (s, 1 H), 7.23–7.30 (m, 2 H), 7.39–7.42 (m, 1 H), 8.22 (s, 1 H), 9.89 (s, 1 H), 11.49 (s, 1 H).

Supporting Methods S4. Immunoblot. Cells were lysed in NP-40 lysis buffer (1% NP40, 20 mM Tris pH 7.9, 150 mM NaCl, 2 mM EDTA+COMPLETE protease inhibitors) and lysates were collected and spun down. Cleared lysates were diluted in 4x sample buffer (Novex) + DTT and boiled for 5 min. The samples were run in a Bis-Tris NuPAGE gel (4-12%) at 120V in 1x NuPAGE MOPS SDS running buffer Soak gel for 10-20 min. Proteins were transferred from gel to membrane in transfer buffer using an iBLOT machine (Thermo Fisher) for 10 min. The membrane was blocked in TBS + 5% milk[a] for 60 min at RT. The primary antibodies anti-EGFR (H9B4 clone, mouse monoclonal), anti-EGFR (5B7 clone, rabbit monoclonal), used to detect EGFR and anti beta-tubulin (rabbit polyclonal, Abcam) were diluted in 5% milk[a] in TBS-T (0.1% Tween-20) (blocking buffer) and incubated at the concentration of 1 μg/ml (or 1:1000 dilution) at 4°C overnight. After overnight incubation the membrane was washed three times for 20 min in TBS-T (0.1% Tween-20) at RT and incubated with fluorescence-labeled secondary antibodies in TBS-T (0.1% Tween-20) for 60 min at RT. The membrane was washed for 3 x 20 min TBS-T (0.1% Tween-20) and further washed for 2 x 5 min in TBS. All the immunoblotting membrane images were recorded using an Odyssey Scanner (LI-COR Bioscience System).

Supporting Method Table ST4. Predicted and observed daughter ions in MS analysis.

		**OligoA1 (Mw: 10147.8)**	**Dasatinib–OligoA1 Mw: 10629.8)**	**Gefitinib–OligoA1 (Mw: 10546.9)**	**Erlotinib–OligoA1 Mw: 10541.0)**
	**x**	**Predicted ions (M-xH)**	**Predicted ions (M-xH)**	**Predicted ions (M-xH)**	**Predicted ions (M-xH)**
1	-1	10146.8	10628.7	10545.9	10540.0
2	-2	5072.9	5313.8	5272.4	5269.5
3	-3	3381.6	3542.2	35146	3512.7
4	-4	2535.9	2656.4	2635.7	2634.2
5	-5	2028.6	2124.9	2108.4	2107.2
6	-6	1690.3	1770.6	1756.8	1755.8
7	-7	1448.7	1517.5	1505.7	1504.9
8	-8	1267.5	1327.7	1317.4	1316.6
9	-9	1126.5	1180.0	1170.9	1170.2
10	-10	1013.8	1061.9	1053.7	1053.1
11	-11	921.5	965.3	957.8	957.3
12	-12	844.6	884.8	877.9	877.4
13	-13	779.6	816.6	810.3	809.8
14	-14	723.8	758.2	752.3	751.9
15	-15	675.5	707.6	702.1	701.7

		**OligoB1 (Mw: 11079.4)**	**Dasatinib–OligoB1 Mw: 10629.8)**	**Gefitinib–OligoB1 (Mw: 11478.5)**	**Erlotinib–OligoB1 Mw: 11472.6)**
	**x**	**Predicted ions (M-xH)**	**Predicted ions (M-xH)**	**Predicted ions (M-xH)**	**Predicted ions (M-xH)**
1	-1	11078.4	10628.7	11477.5	11471.6
2	-2	5538.7	5313.8	5738.3	5735.3
3	-3	3692.1	3542.2	3825.2	3823.2
4	-4	2768.9	2656.4	2868.6	2867.2
5	-5	2214.9	2124.9	2294.7	2293.5
6	-6	1845.6	1770.6	1912.1	1911.1
7	-7	1581.8	1517.5	1638.8	1637.9
8	-8	1383.9	1327.7	1433.8	1433.1
9	-9	1230.0	1180.0	1274.4	1273.7
10	-10	1106.9	1061.9	1146.9	1146.3
11	-11	1006.2	965.3	1042.5	1042.0
12	-12	922.3	884.8	955.5	955.1
13	-13	851.3	816.6	882.0	881.5
14	-14	790.4	758.2	818.9	818.5
15	-15	737.6	707.6	764.2	763.8

Supporting Table ST1. Recombinant human proteins used for validation of drug binding

Protein	Catalogue Number	Suppliers	Molecular Mass (MW)	Application	Nature of protein
ABL1	P3049	Life Technologies	125.4 kDa	Kinase assay	Full-length histidine-tagged protein
EGFR-L858R	PV4128	ThermoFisher	90.5 kDa	Kinase assay	EGFR L858R pint mutant recombinant human Protein

Kinase activity, e.g. :- LanthaScreen Kinase Activity Assays

Supporting Materials Table ST2. Antibodies used in this study.

Antibody	Catalogue number	Suppliers	Source & clonality (IgG)	Applications*	Epitope/specificity
EGFR Ab-15 (H9B4)	MS-665-PABX	Fisher Scientific	Mouse monoclonal	WB, IP	Cytoplasmic domain
CONFIRM™ EGFR (3C6 or 5B7)	790-4347	Ventana Medical Systems	Rabbit monoclonal	IHC, IVD	Intracellular domain of EGFR
c-ABL	AF5414	RnD Systems	Goat polyclonal	WB	Ala941-Val1140 at the C-terminus
BCR	AF5129	RnD Systems	Sheep polyclonal	WB	Lys174-Asp331 at the N-terminus
beta-tubulin	ab6046	Abcam	Rabbit polyclonal	WB, IP, IF, IHC, ELISA, ICC	Residues 1 - 100 of human beta Tubulin
6xHIS tag	Ab18184	Abcam	Mouse monoclonal	WB, IP, IF, IHC, ELISA, ICC	His –tagged proteins
GST	D9-1310	Columbia Bioscience	Goat polyclonal	IF, IHC, ELISA,	IgG conjugated to DyLight® 550

WB (western blot), ELISA (enzyme-linked immunosorbent assay), FC (flow cytometry) IF (immunofluorescence) ICC (immunocytochemistry), IHC (immunohistochemistry), IP (immunoprecipitation), IVD (In vitro diagnostic)

Supporting Table ST3. Kinase inhibitors, structure and target kinases(5-10).

Compound*, other names	Development status	Structure	Structure of analogue (precursor compound)	*Known targets for original compound
Gefitinib, ZD-1839; Iressa® (Astra Zeneca)	Marketed drug	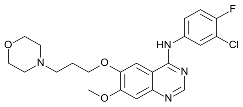	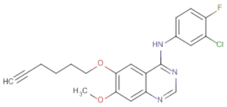	EGFR, ABL1, CSNK1E, EPHA6, ERBB1, ERBB2, ERBB4, GAK, JNK2, JNK3, LCK, LOK, MKNK2, RIPK2, SLK, SRC, STK17A
Dasatinib (Sprycel)	Marketed drug	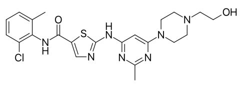	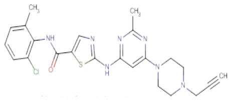	ABL1, ABL2, BCR-ABL, SRC, DDR1&2, EGFR, EPHA8, LCK, FGR, FRK, FYN, KIT, MAPK14, PDGFRA&B
Erlotinib, (Tarceva)	Marketed drug	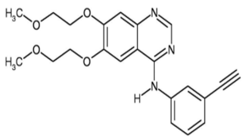	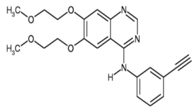	EGFR, ABL1, ABL2, EPHA6, ERBB2, ERBB4, GAK, LCK, LOK, JNK2, MKNK2, RIPK2, SLK, SRC
Bosutinib	Marketed drug	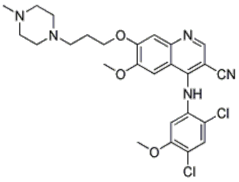		ABL1, ABL2, BCR, BCR-ABL1, BCR-ABL2, BTK, BMX, BTK, CSK, EPHB4, FER, FRK, FGR, FYN, HCK, LCK, LYN, MAP4K2, MAP4K5, PDGFRA, PTK2, SRC, STK24, TEC, YES1, TNK2, ZAK
Staurosporine	Research tool	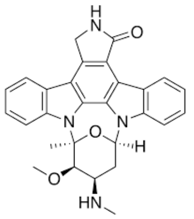		pan-kinase, PRKCH, EGFR, ABL1, ABL2, CSNK1E, EPHA6, EPHA8, ERBB1, FRK, FYN, GAK, JNK1-3, KIT, LCK, LOK, MKNK2, RIPK2, SLK, SRC, STK17A, PDGFRB, VEGFR2
Ibrutinib	Marketed drug	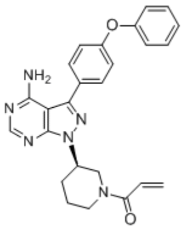		ABL1, ABL2, BLK, BMX, CSK, EGFR, ErbB2, FRG, FLT3, FRK, FYN, HCK, JAK3, LCK, LYN, MAPK3K1, PDGFRA, PTK6, RET, RIPK2, SRC, TEC, YES1

REFERENCES

1. Li, Z., Hao, P., Li, L., Tan, C. Y., Cheng, X., Chen, G. Y., Sze, S. K., Shen, H. M., & Yao, S. Q. (2013) Design and synthesis of minimalist terminal alkyne-containing diazirine photo-crosslinkers and their incorporation into kinase inhibitors for cell- and tissue-based proteome profiling. *Angew. Chem. Int. Ed. Engl.*, **52**, 8551-8556.

2. Liu, Y.; Gray, N. S. (2006) Rational design of inhibitors that bind to inactive kinase conformations. *Nat. Chem. Biol.*, **2**, 358-364.

3. Dietrich, J.; Hulme, C.; Hurley, L. H. (2010) The design, synthesis, and evaluation of 8 hybrid DFG-out allosteric kinase inhibitors: A structural analysis of the binding interactions of Gleevec®, Nexavar®, and BIRB-796. *Bioorg. & Medicinal Chem.*, **18**, 5738-5748.

4. Parker, J. J.; Dionne, K. R.; Massarwa, R.; Klaassen, M.; Foreman, N. K.; Niswander, L.; Canoll, P.; Kleinschmidt-Demasters, B. K.; Waziri, A. (2013) Gefitinib selectively inhibits tumor cell migration in EGFR-amplified human glioblastoma. *Neuro-Onco.*, **15**, 1048-1057.

5. Chidley, C.; Haruki, H.; Pedersen, M. G.; Muller, E.; Johnsson, K. (2011) A yeast-based screen reveals that sulfasalazine inhibits tetrahydrobiopterin biosynthesis. *Nat. Chem. Biol.*, **7**, 375-383.

6. Fabian, M. A.; Biggs, W. H.; 3rd, Treiber, D. K.; Atteridge, C. E.; Azimioara, M. D.; Benedetti, M. G.; Carter, T. A.; Ciceri, P.; Edeen, P. T.; Floyd, M.; Ford, J. M.; Galvin, M.; Gerlach, J. L.; Grotzfeld, R. M.; Herrgard, S.; Insko, D. E.; Insko, M. A.; Lai, A. G.; Lélias, J. M.; Mehta, S. A.; … Lockhart, D. J. (2005) A small molecule-kinase interaction map for clinical kinase inhibitors. *Nat. Biotechnol.*, **23**, 329-336.

7. Davis, M. I.; Hunt, J. P.; Herrgard, S.; Ciceri, P.; Wodicka, L. M.; Pallares, G.; Hocker, M.; Treiber, D. K.; Zarrinkar, P. P. (2011) Comprehensive analysis of kinase inhibitor selectivity. *Nat. Biotechnol.*, **29**, 1046-1051.

8. Bantscheff, M.; Eberhard, D.; Abraham, Y.; Bastuck, S.; Boesche, M.; Hobson, S.; Mathieson, T.; Perrin, J.; Raida, M.; Rau, C.; Reader, V.; Sweetman, G.; Bauer, A.; Bouwmeester, T.; Hopf, C.; Kruse, U.; Neubauer, G.; Ramsden, N.; Rick, J.; Kuster, B.; … Drewes, G. (2007) Quantitative chemical proteomics reveals mechanisms of action of clinical ABL kinase inhibitors. *Nat. Biotechnol.*, **25**, 1035–1044.

9. Kitagawa, D.; Yokota, K.; Gouda, M.; Narumi, Y.; Ohmoto, H.; Nishiwaki, E.; Akita, K.; Kirii, Y. (2013) Activity-based kinase profiling of approved tyrosine kinase inhibitors. *Genes to cells*, **18**, 110–122.

10. Anastassiadis, T.; Deacon, S. W.; Devarajan, K.; Ma, H.; Peterson, J. R. (2011) Comprehensive assay of kinase catalytic activity reveals features of kinase inhibitor selectivity. *Nat. Biotechnol.*, **29**, 1039–1045.
